# Effects of Rearing Conditions on Behaviour and Endogenous Opioids in Rats with Alcohol Access during Adolescence

**DOI:** 10.1371/journal.pone.0076591

**Published:** 2013-10-02

**Authors:** Sara Palm, Loudin Daoura, Erika Roman, Ingrid Nylander

**Affiliations:** Neuropharmacology, Addiction & Behaviour, Department of Pharmaceutical Biosciences, Uppsala University, Uppsala, Sweden; Radboud University, Netherlands

## Abstract

Causal links between early-life stress, genes and later psychiatric diagnoses are not possible to fully address in human studies. Animal models therefore provide an important complement in which conditions can be well controlled and are here used to study and distinguish effects of early-life stress and alcohol exposure. The objective of this study was to investigate the impact of rearing conditions on behaviour in young rats and if these changes could be followed over time and to examine interaction effects between early-life environment and adolescent alcohol drinking on behaviour and immunoreactive levels of the opioid peptides dynorphin B, met-enkephalin-Arg^6^Phe^7^ and beta-endorphin. We employed a rodent model, maternal separation, to study the impact of rearing conditions on behaviour, voluntary alcohol consumption and alcohol-induced effects. The consequences of short, 15 min (MS 15), and long, 360 min (MS 360), maternal separation in combination with adolescent voluntary alcohol consumption on behaviour and peptides were examined. A difference in the development of risk taking behaviour was found between the MS15 and MS360 while the development of general activity was found to differ between intake groups. Beta-endorphin levels in the pituitary and the periaqueductal gray area was found to be higher in the MS15 than the MS360. Adolescent drinking resulted in higher dynorphin B levels in the hippocampus and higher met-enkephalin-Arg^6^Phe^7^ levels in the amygdala. Amygdala and hippocampus are involved in addiction processes and changes in these brain areas after adolescent alcohol drinking may have consequences for cognitive function and drug consumption behaviour in adulthood. The study shows that individual behavioural profiling over time in combination with neurobiological investigations provides means for studies of causality between early-life stress, behaviour and vulnerability to psychiatric disorders.

## Introduction

The individual genetic make-up combined with the environment, particularly early in life, have a large impact on the risk of developing later psychiatric illness [1-3], for example alcohol use disorders (AUD) [4,5]. Gene × environment interactions can also affect the response to treatment [6,7]. In search for better, personalised prevention and treatment strategies it is important to investigate the underlying factors leading to the adult phenotype, which in AUD includes cognitive dysfunction and compulsive alcohol intake. Most individuals are exposed to alcohol during adolescence when the brain undergoes extensive development and maturation [8,9]. The combination of alcohol intake and social environment during this sensitive time window can have detrimental effects on the individual and it is therefore vital to increase our knowledge about the consequences of early-life adversity. However, causal relations between environmental factors, genes, phenotypes and AUD are complicated and not possible to fully address in studies of humans. Certain behavioural phenotypes have for example been linked to a propensity for alcohol intake [10-12]. However, it is difficult to determine if a specific phenotype is the cause of the excessive drinking or if the phenotype is a consequence of early-life drinking. Therefore, animal models with controlled conditions are important complements that give us insight in neurobiological [13,14] and behavioural [15,16] consequences of adolescent alcohol exposure. Experimental studies can provide information about whether proneness to AUD is inherent, a consequence of early-life adversity, adolescent alcohol exposure or a combination of these factors.

Early-life adversity is currently studied in a rodent experimental model, maternal separation (MS), in which the impact of rearing conditions on behaviour, voluntary alcohol consumption and alcohol-induced effects can be investigated. The MS model allows the simulation of a risk environment with longer separations (180-360 min; MS180-360) than usually seen in a natural environment where the dam leaves her litter for shorter time periods (15 min; simulated by MS15) [17,18]. Exposure to prolonged separations provides means to study effects induced by interruption of the normal mother-pup interactions [19,20].

Longer separation periods result in rats that, in adulthood, drink more alcohol [21-23], increase their alcohol preference over time [24] and have higher preference for 20% alcohol concentration [25] than rats exposed to shorter duration of separation. The well-described link between the endogenous opioid system and alcohol [26,27] raises the question whether changes in the opioid system by MS could be one of the underlying factors behind the differences in adult drinking seen between the rats exposed to different separation durations. For example, opioids have been implicated in the mechanism of action of alcohol [28,29] and in propensity for AUD [26,30,31]. The effectiveness of opioid antagonists to reduce alcohol intake in both animals and humans further supports opioid involvement [32-36]. Previous data from our group and others show interesting differences in opioid peptide content in several rat brain areas [37], both after MS alone [38,39], and in combination with adult alcohol drinking [40]. Differences in the response to the opioid antagonist naltrexone [41] and to an opioid agonist [42] have also been shown. These data together with the involvement of opioids in early life social behaviour [43-47] indicate a possible link between early life environment, opioids and adult drinking behaviour.

However, a recent study revealed that adolescent drinking was not dependent on early-life rearing conditions [24] and the question of whether this would persist into adulthood or not arose. It was also of interest to examine whether adolescent drinking would elicit different alcohol-induced effects depending on rearing environment. We hypothesized that MS followed by adolescent alcohol intake may result in similar alcohol intake in adulthood, but the alcohol-induced effects on opioids may differ. We here extend our previous studies to include the consequences of MS in combination with adolescent voluntary alcohol consumption on the development of behaviour, adult alcohol intake and endogenous opioid peptides. Information about the MS-induced effects on behaviour and how adolescent drinking modulates the behavioural development could provide important clues as to what comes first, the behaviour or the intake, and how the two can interact.

The overall aim was to investigate the impact of early-life conditions in combination with adolescent alcohol intake on behavioural development and endogenous opioids. The multivariate concentric square field™ (MCSF) test was used to profile individual behaviour. Since young rats had not been previously profiled in this test, it was important to investigate how a regular laboratory rat performs in this test at this age. Therefore, animal facility reared (AFR) rats, were included to serve as a behavioural control to the MS groups. The first objective was therefore to investigate whether the early-life environment would affect behaviour in young rats and if these changes could be followed over time by testing them again in adulthood.

The second objective of the study was to examine interactions between MS and voluntary adolescent drinking on behavioural development and on endogenous opioids. To determine whether MS leads to behavioural differences at an early age and if the behaviour could be differentially modulated over time by adolescent alcohol intake was the MCSF test was performed before and after adolescent alcohol access. In the neurochemical analysis it was investigated whether changes in the opioid system occurred and if these were dependent on the rearing environment, the adolescent alcohol intake or a combination of the two. The levels of the opioid peptides dynorphin B (DYNB), met-enkephalin-Arg^6^Phe^7^ (MEAP) and beta-endorphin (BEND) were analysed in the pituitary gland and several brain areas.

## Materials and Methods

### Ethics statement

All animal experiments were performed under a protocol approved by The Uppsala Animal Ethical Committee and followed the guidelines of the Swedish Legislation on Animal Experimentation (Animal Welfare Act SFS1998:56) and The European Communities Council Directive (86/609/EEC).

### Animals

Pregnant Wistar Han (RccHan:WI) rats (Harlan Laboratories B.V., Horst, the Netherlands) arrived at the animal facility on gestation day 15-16. The dams were kept in cages type IV (59 x 38 x 20 cm) containing wood chip bedding and paper towels, in temperature- (22±1 °C) and humidity- (50±10%) controlled housing cabinets in a room with a 12 h light/dark cycle, with lights on at 07:00 h. All animals had *ad libitum* access to pellet food (Type R36; Lantmännen, Kimstad, Sweden) and water.

### Rearing conditions

**Figure 1:**
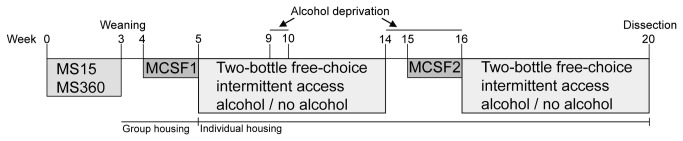
An overview of the experimental outline. MS15 = maternal separation 15 min, MS360 = maternal separation 360 min, MCSF = multivariate concentric square field™ test; w = weeks of age.

The litters that were born on the same day (postnatal day (PND) 0) were cross-fostered to include 5-6 males and 4-5 females. Figure 1 gives an overview of the experimental procedure. The litters were randomly assigned to one of two rearing conditions during PND 1-21: 1) daily 15 min maternal separations (MS15, n = 5 litters), or 2) daily 360 min maternal separations (MS360, n = 5 litters). During the separations the litters were kept together in cages type II (26 x 20 x 14 cm) containing wood chip bedding and held in a heating cabinet (30.5±1 °C) to avoid hypothermia. Separation sessions were performed during the light period of the light/dark cycle with 15 min separations starting at 09:00 h and 360 min separations starting at 09:30 h. The MS15 and MS360 litters were weighed at PND 1, 4, 7, 10, 13, 16 and 19. For the purpose of controlling for the testing of young animals in the behavioural test, another group of rats, animal facility reared (AFR), was also included in this part of the study. The AFR litters were left undisturbed and only handled when weighed once a week on PND 1, 7 and 13.

The pups were weaned on PND 22 and housed 5-6 per cage until PND 34. Only male pups were further used in this study. On the day of weaning the male rats were moved to a room with reversed light/dark cycle, with lights off at 09:30 h.

### Behavioural analysis

All animals (AFR, n = 10; MS15, n = 30; MS360, n = 30) were tested in a 20 min trial in the multivariate concentric square field™ (MCSF) test (100 x 100 cm) at two occasions, PND 27-31 and PND 104-108. The second test was performed during the alcohol deprivation period to avoid alcohol on board in the alcohol-drinking rats. Repeated testing of animals during development in the MCSF has not been done previously and AFR rats therefore served as controls for the behavioural development in a regular laboratory rat, as measured by the MCSF test. This test is an ethologically founded test and unlike many other common tests, the MCSF test is unprejudiced with regard to mental condition allowing for a more diverse behavioural profile. The MCSF test arena, the general testing procedure and the behavioural recording have been described in detail elsewhere [48,49]. The entire arena is divided into zones, which forms the basis of the description and the variables of the animals’ performance in this test. An operational categorization of the various parameters with regard to function (i.e. general activity, exploratory activity, risk assessment, risk taking and shelter seeking) is used in the interpretation of results [49].

### Alcohol drinking paradigm

On PND 34 all rats were individually housed in cages type III (42 x 26 x 18 cm) containing wood chip bedding and a wooden house. All animals had *ad libitum* access to pellet food (Type R36; Lantmännen, Kimstad, Sweden) and water. The MS rats were randomly assigned to drink ethanol (E) or water (W) (MS15W; n = 10, MS15E; n = 20, MS360W, n = 10; MS360E, n = 20). The alcohol access was a two-bottle free choice paradigm with 24 h intermittent access to 20% ethanol on Mondays, Wednesdays and Fridays. Bottle positions were switched between sessions to avoid position preference. During sessions, water control rats also had access to two bottles, with tap water only. Intake was measured at the end of the 24 h access period by weighing the bottles. Alcohol preference was measured as the percentage of the total intake that was from the ethanol bottle. On the days in between alcohol access, one water bottle was available. Alcohol solutions were made from 96% ethanol (Solveco Etanol A 96%; Solveco AB, Rosersberg, Sweden) and tap water. Sessions were initiated on PND 34 and lasted until the animals were 20 weeks old. Alcohol deprivation periods were introduced during sessions 13-15 and sessions 27-32, Figure 1. During these sessions only water was accessible to the animals. In total, the rats had alcohol access for 12 weeks (36 sessions).

### Dissection

At twenty weeks of age, the rats were decapitated immediately after the last alcohol session. The pituitary gland was divided into the neurointermediate (NIL) and anterior lobes. The hypothalamus was removed from the brain, which was then placed in a cooled matrix (ASI Instruments, Inc., Warren, MI) and from coronal sections frontal cortex, medial prefrontal cortex, nucleus accumbens, dorsal striatum, hippocampus, amygdala, substantia nigra, ventral tegmental area and periaqueductal grey (PAG) were dissected. All tissues were immediately frozen on dry ice and stored in -80 °C.

### Peptide analysis

Previously descried protocols were used for tissue extraction [50] and radioimmunoassays (RIAs) [51]. For the BEND assay rabbit-anti-beta-endorphin serum (Peninsula Laboratories LLC, San Carlos, CA) was used. Antibody bound DYNB and BEND was separated from free peptides by adding 50 µl goat-anti-rabbit-IgG and 50 µl normal rabbit serum (Peninsula Laboratories LLC, San Carlos, CA) whereas for the MEAP assay, a charcoal suspension was added.

### Statistical analysis

Statistical analyses were performed using Statistica 9.1 (StatSoft Inc., Tulsa, OK). Differences were considered statistically significant at *p* < 0.05. For the multivariate data analysis, SIMCA-P+ 12.0 (Umetrics AB, Umeå, Sweden) was used.

#### Data handling

For analysis of descriptive behavioural parameters, the data was logarithmically transformed (log (x+1)) to achieve a normal distribution and analysed using parametric statistics. In the trend analysis, see 49,52 for further details, behavioural parameters for each individual were ranked, i.e. the animal with the lowest descriptive score is given the lowest rank value and vice versa. The rank values are then summed into functional categories; general activity (total activity, frequency and duration/frequency in all corridors and frequency in centre), exploratory activity (duration in all corridors, centre and hurdle, rearing and photocell counts on hurdle), risk assessment (duration/frequency on slope and bridge entrance and stretch attend postures to centre), risk taking (frequency, duration and duration/frequency on the bridge and in the central circle) and shelter seeking (frequency, duration and duration/frequency in the dark corner room), which allowed for the use of parametric statistics.

#### Effects of different rearing conditions

The impact of rearing condition on behaviour in the 4-week-old rats was analysed using one-way analysis of variance (ANOVA) between all AFR, MS15 and MS360. Analysis of the impact of rearing condition on behavioural change over time was done by repeated measures ANOVA in water-drinking AFR, MS15 and MS360 rats. Post-hoc analysis was performed with the Fisher’s least significant difference (LSD) test.

#### Effects on voluntary alcohol consumption

Alcohol intake was not normally distributed, as tested by the Shapiro-Wilk’s test, and was therefore assessed using non-parametric statistics. Differences in fluid intake between the two MS groups were analysed using the Mann-Whitney *U*-test and changes over time for the respective groups were assessed using the Friedman test followed by the Wilcoxon matched-pairs signed-rank test.

#### Interactive effects of maternal separation and adolescent voluntary drinking

Repeated measures ANOVA with MS group (MS15 or MS360) and intake group (E or W) as factors were used to analyse behaviour after different MS conditions. In this part of the study, we considered the MS15 to be an appropriate control for the MS360 and the AFR rats were left out, mainly due to the debate in the literature of whether this group is a proper control to prolonged MS [19,53].

Factorial ANOVA with MS group and intake group as factors was used to analyse alcohol-induced effects on opioids in rats subjected to different MS conditions. Post-hoc analysis was performed with the Fisher’s LSD test.

#### Relationship between behaviour and alcohol intake

Multivariate data analysis with principal component analysis (PCA) and partial least squares projection to latent structures (PLS) was used to investigate the relationship between behavioural parameters and alcohol intake.

## Results

### Impact of rearing conditions on behaviour

**Figure 2:**
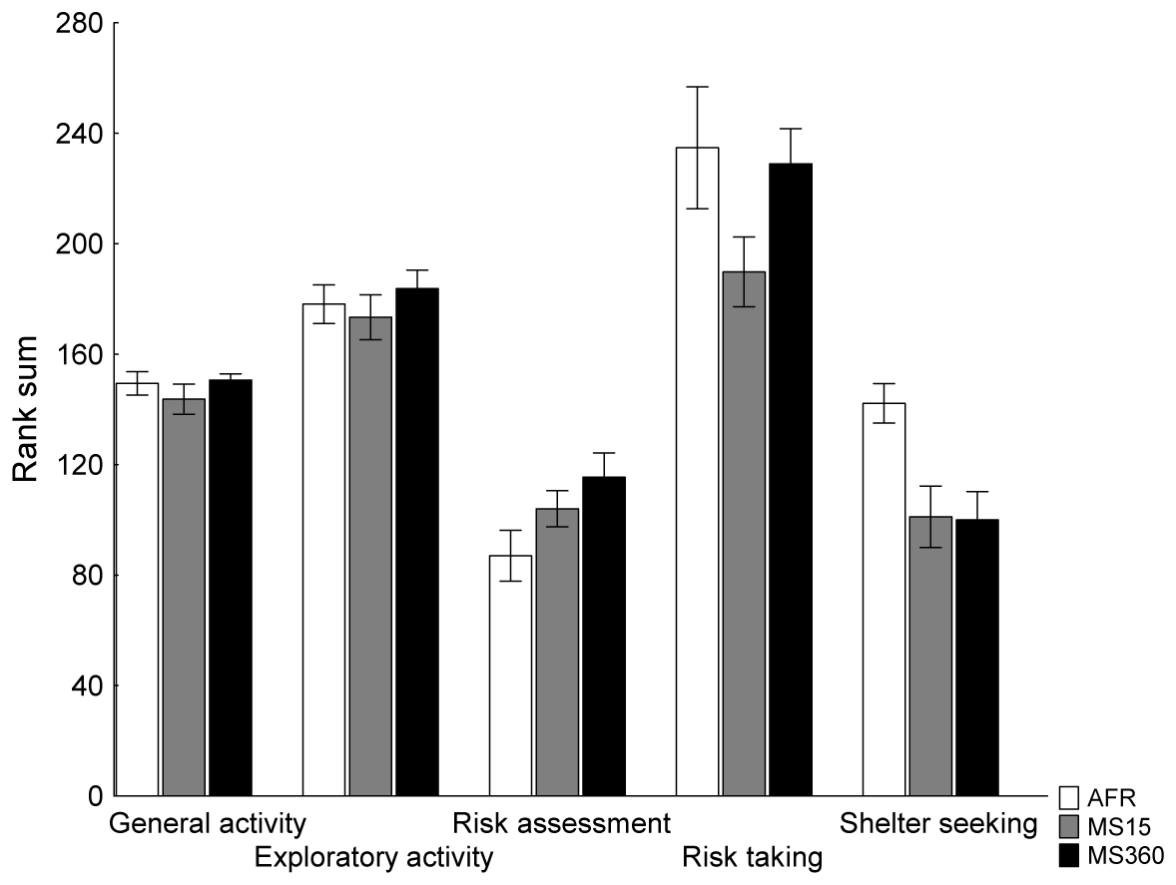
Trend analysis of the first multivariate concentric square field™ test for all rats at 4 weeks of age. Data are expressed as mean ± SEM. AFR = animal facility reared, MS15 = maternal separation 15 min, MS360 = maternal separation 360 min.

In the trend analysis of all 4-week-old rats, using one-way ANOVA, no statistically significant differences were found, Figure 2. There was however, a tendency towards differences in risk taking [F(2, 67)=532; p=0.06] driven by the MS15 that displayed less risk taking behaviour than the other groups. There was also a tendency in shelter seeking behaviour [F(2, 67)=232; p=0.09] where the MS15 and MS360 rats displayed less shelter seeking behaviour than the AFR rats. The classical statistics for the descriptive behavioural parameters behind the trend analysis can be found in Table S1.

**Figure 3:**
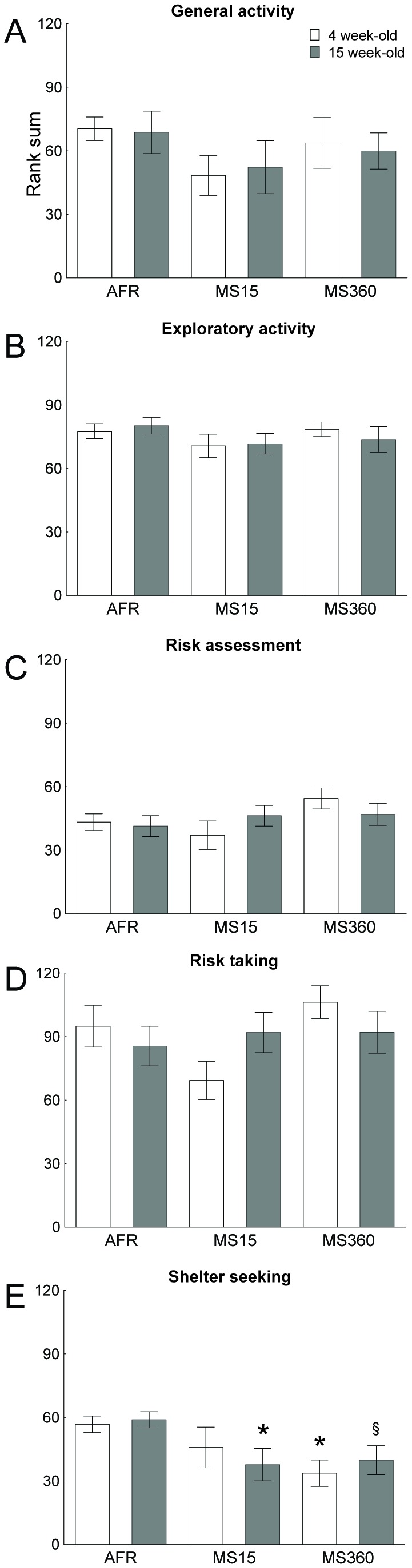
Trend analysis of the two multivariate concentric square field™ tests in the three rearing groups. The analysis was made at 4 and 15 weeks of age in water-drinking animals from the three rearing groups: animal facility reared (AFR), maternal separation 15 min (MS 15) and maternal separation 360 min (MS 360). Each graph shows one of the functional categories: **a**) general activity, **b**) exploratory activity, **c**) risk assessment, **d**) risk taking, and **e**) shelter seeking. Data are expressed as mean ± SEM. * *p* < 0.05 compared to same age AFR, ^§^ *p* < 0.06 compared to same age AFR, (repeated measures ANOVA followed by Fisher’s LSD test).

In the adult water-drinking rats, few differences were found. Figure 3 summarizes the differences between the water-drinking AFR, MS15 and MS360 rats in the trend analysis at 4 and 15 weeks of age, using repeated measures ANOVA, in the categories general activity (Figure 3a), exploratory activity (Figure 3b), risk assessment (Figure 3c), risk taking (Figure 3d) and shelter seeking (Figure 3e). The ANOVA displayed a main effect of group [F(2, 26)=3.61; p=0.04] on shelter seeking behaviour, which according to the post-hoc test was due to the young MS360 rats displaying less shelter seeking behaviour compared to the young AFR rats (p=0.02) and there was a strong trend towards a similar difference in the adult rats (p=0.06), Figure 3e. In adulthood, the MS15 also differed in shelter seeking behaviour compared to AFR (p=0.04).

### Interaction effects of maternal separation and adolescent alcohol drinking

#### Alcohol intake

No differences in alcohol intake were found between the MS groups at any time point, see Table 1, and the groups did not significantly change their intake over time as analysed by the Friedman test for MS15 [χ^2^=16.2; p=0.13] and MS360 [χ^2^=6.10; p=0.87]. In addition, no increase in alcohol intake due to deprivation of alcohol, i.e. alcohol deprivation effect, analysed by Wilcoxon matched-pairs test, was found after the first one-week [MS15 Z=1.19; p=0.23 and MS360 Z=0.75; p=0.46] or the later two-week deprivation period [MS15 Z=0.07; p=0.94 and MS360 Z=0.36; p=0.74]. Alcohol preference was not different between the two MS groups, but both groups increased their preference significantly over time from about 12% to about 30% [MS15 χ^2^=105; p<0.001 and MS360 χ^2^=111; p<0.001], Figure S1. No differences in water intake or total fluid intake were found (data not shown).

**Table 1:** The weekly median, minimum and maximum ethanol intake (g/kg/day) in the two MS groups during the weeks of intermittent ethanol access.

	**MS15**			**MS360**			
**Age (weeks)**	**Ethanol (g/kg/day)**	**Min**	**Max**	**Ethanol (g/kg/day)**	**Min**	**Max**	**Mann-Whitney U test**
5	3.3	2.7	4.2	3.0	2.2	9.1	*Z* = 1.3; *p* = 0.18
6	2.9	2.1	8.8	2.6	2.2	4.6	*Z* = 1.3; *p* = 0.20
7	2.9	1.6	7.9	2.6	1.7	4.9	*Z* = 0.6; *p* = 0.64
8	2.8	1.6	8.4	2.5	1.3	6.7	*Z* = 0.9; *p* = 0.36
9	Alcohol deprivation						
10	2.7	1.5	6.4	2.3	1.3	4.3	*Z* = 1.2; *p* = 0.22
11	2.8	1.6	6.0	2.5	1.6	4.7	*Z* = 1.3; *p* = 0.20
12	2.8	1.4	6.0	2.5	1.7	4.7	*Z* = 0.7; *p* = 0.51
13	2.7	1.2	6.0	2.5	1.5	6.2	*Z* = 0.1; *p* = 0.90
14	Alcohol deprivation						
15	Alcohol deprivation						
16	2.9	0.9	6.0	2.8	1.3	6.0	*Z* = 0.0; *p* = 0.97
17	2.8	0.9	5.9	2.6	1.2	5.2	*Z* = -0.1; *p* = 0.88
18	3.0	1.1	6.1	2.9	1.4	5.1	*Z* = -0.3; *p* = 0.78
19	2.7	1.0	5.4	2.8	1.5	5.0	*Z* = 0.2; *p* = 0.82

MS15 = maternal separation 15 min, MS360 = maternal separation 360 min

#### Behaviour

**Figure 4:**
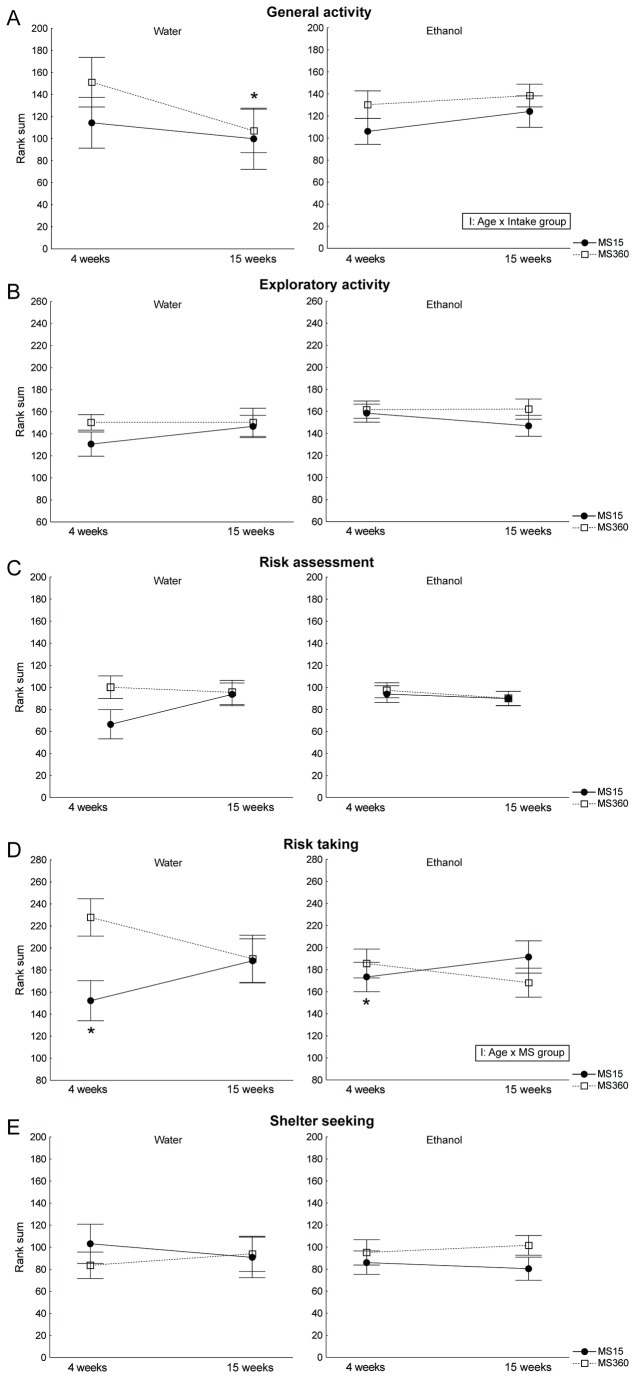
Trend analysis of the two MCSF tests in maternal separation animals. The analysis was made at 4 and 15 weeks of age in the ethanol (E) and water (W) groups of the maternal separation 15 min (MS 15) and maternal separation 360 min (MS 360). Each graph shows one of the functional categories: **a**) general activity, **b**) exploratory activity, **c**) risk assessment, **d**) risk taking, and **e**) shelter seeking. Note that at age 4 weeks no animals had access to alcohol, but were only designated to a drinking paradigm. Data are expressed as mean ± SEM. * *p* < 0.05 compared to water-drinking MS360 at 4 weeks of age, I = interaction effect (repeated measures ANOVA followed by Fisher’s LSD test).

Figure 4 summarizes behaviour over time in water-drinking rats as well as alcohol-induced effects in the two MS groups. The repeated measures ANOVA of the trend analysis revealed a significant interaction between age and intake group in the category general activity [F(1, 56)=6.87; p=0.01], Figure 4a. Overall, the water-drinking rats decreased their general activity over time (p=0.03), while the alcohol-drinking rats remained the same, and this effect was mainly due to the decrease in the water-drinking MS360 group (p=0.02). Interaction effects between age and MS group were found in risk taking [F(1, 56)=6.35; p=0.01], Figure 4d. The MS15 group displayed lower risk taking than the MS360 group at 4 weeks of age (p=0.04), but at 15 weeks of age these differences were diminished. The differences summarized in the trend analysis are also reflected in the classical statistics for each descriptive parameter and can be found in Table S1.

#### Relationship between behaviour and alcohol intake

The PLS model of behaviour at 4 weeks of age did not significantly explain the initiation of alcohol intake measured as intake during any of the first three sessions (no significant components [R^2^_X_=0.12, R^2^_Y_=0.17, Q^2^=-0.12] for first component) or the mean intake during the first week of alcohol access (no significant components, [R^2^_X_=0.14, R^2^_Y_=0.32, Q^2^=-0.21] for first component), nor did it explain any of the following weeks of intake (no significant components, [R^2^_X_=0.16, R^2^_Y_=0.09, Q^2^=-0.15] for first component). The alcohol intake between behavioural test 1 and 2 did not significantly explain the behaviour during test 2 (no significant components, [R^2^_X_=0.30, R^2^_Y_=0.04, Q^2^=-0.04] for first component).

#### Peptide levels

**Figure 5:**
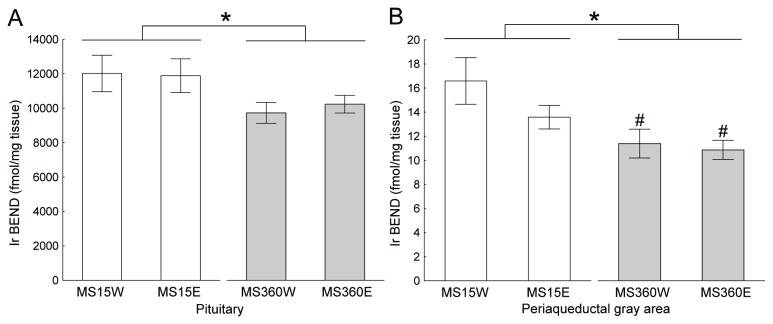
Rearing-induced beta-endorphin (BEND) levels. The bars show the difference between groups of the maternal separation 15 min (MS 15) and maternal separation 360 min (MS 360) in mean ± SEM levels of beta-endorphin in **a**) the pituitary and **b**) the periaqueductal gray area. Ir = immunoreactive, E = ethanol, W = water, * *p* < 0.05 overall effect of MS group, ^#^ p <0.05 compared to MS15W (factorial ANOVA followed by Fisher’s LSD test).

**Figure 6:**
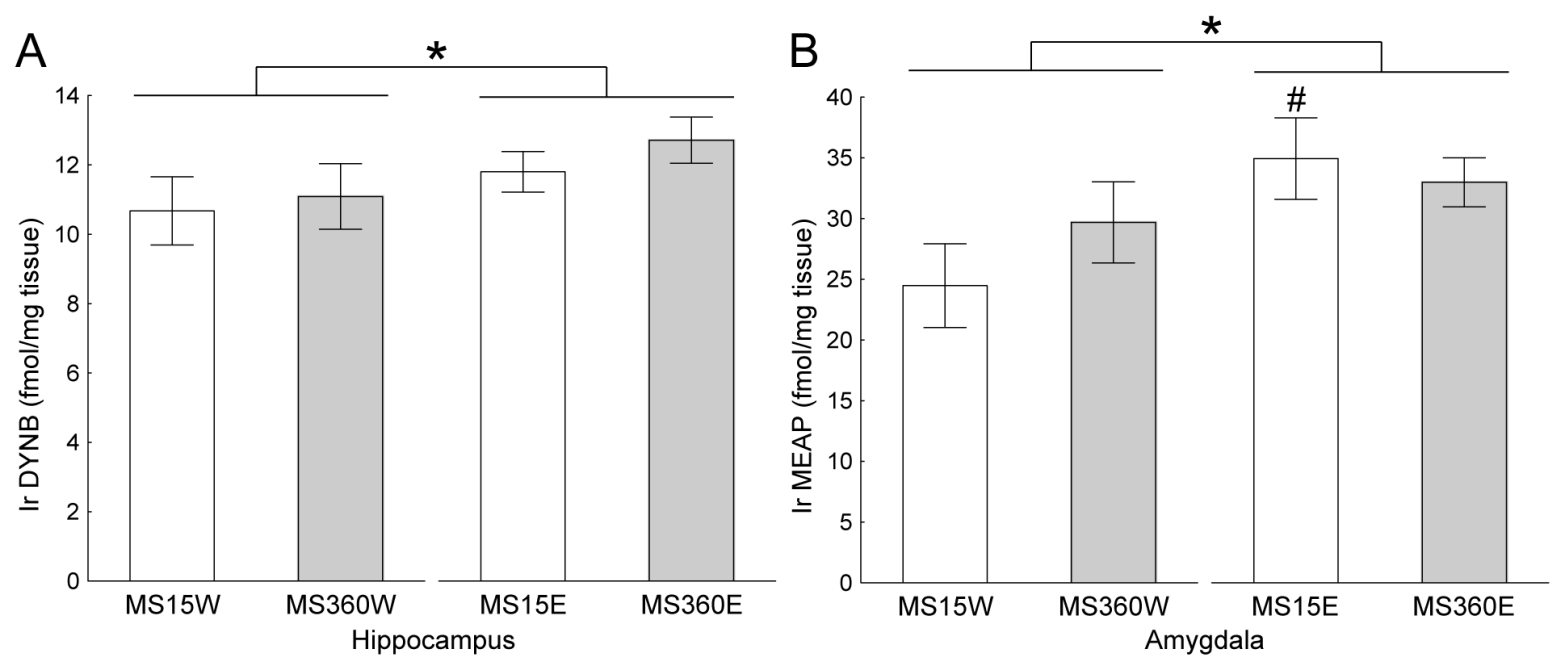
Alcohol-induced peptide levels in the hippocampus and amygdala. The bars show the difference between water (W) and ethanol (E) groups of the maternal separation 15 min (MS 15) and maternal separation 360 min (MS 360) in mean ± SEM levels of **a**) dynorphin B (DYNB) in the hippocampus and **b**) Met-enkephalin-Arg^6^Phe^7^ (MEAP) in the amygdala. Ir = immunoreactive, * *p* < 0.05 overall effect of intake group, ^#^ p <0.05 compared to MS15W (factorial ANOVA followed by Fisher’s LSD test).

In the pituitary and PAG there was a main effect of MS [F(1, 47)=4.10; p=0.048 and F(1, 53)=11.1; p=0.002, respectively]; in both areas the MS360 rats had lower levels of immunoreactive (ir) BEND than the MS15 rats (p=0.03 and p=0.002, respectively), Figure 5a and b. As can be seen in Table S2 the main contribution to the effect in the pituitary comes from the NIL although the effect was only a trend [F(1,47)=3.38; p=0.07] in the NIL alone. In the hypothalamus there was a trend to a main effect of alcohol on ir BEND [F(1, 54)=3.97; p=0.051], with lower levels in the alcohol-drinking rats (p=0.055), Table S2. A main effect of alcohol was found on the levels of ir DYNB in the hippocampus [F(1, 56)=4.23; p=0.04], where alcohol induced an increase of the peptide levels (p=0.04), Figure 6a. No differences in peptide levels between MS-groups were revealed, although there is a trend (p=0.06) towards lower levels in the MS360 groups in the NIL, Table S3. A main effect of alcohol was also found on ir MEAP levels in the amygdala [F(1, 55)=4.34; p=0.04], where alcohol induced an increase of the peptide levels (p=0.04). This effect was statistically significant in the MS15 rats (p=0.03), Figure 6b and Table S4. There were no statistically significant interactions between rearing environment and alcohol drinking on any of the peptide levels measured.

## Discussion

To the best of our knowledge this is the first study to investigate individual behavioural profiles over time in animals exposed to different rearing conditions in combination with the impact of adolescent alcohol drinking on this trajectory as well as on endogenous opioids in adulthood. The main finding was that neurochemical analyses of brain opioid levels showed interesting effects of MS on BEND levels in the pituitary and PAG and of voluntary early alcohol consumption on DYNB levels in hippocampus and MEAP levels in the amygdala. With regard to differences between the MS15, MS360 and AFR rats based on their behaviour at 4 weeks of age only modest effects were revealed and only a few changes over time were dependent on the rearing environment or alcohol intake.

### Impact of rearing conditions on behaviour

The trend analysis of the MCSF test at 4 weeks of age showed modest differences between the groups. There was a tendency for the MS15 to be less risk taking than the AFR and MS360 groups and a tendency for the MS15 and MS360 rats to be less shelter seeking than the AFR rats. Other studies investigating the behaviour of young Wistar rats after MS have not found any differences between MS rats and control groups in for example the open field [54] and this is in agreement with the current study, where there are few differences in the parameters that can be compared to open field parameters, i.e. the centre and central circle. In a study using the elevated plus maze the MS360 had a lower number of open arm entries compared to AFR [55], which could not be seen with the behavioural test used in the current study and may be explained by the fact that the design of the bridge is quite different from an open arm and that the animals tested were only three weeks old. There are also previous data indicating that the animals experience the two risk areas in the MCSF test, i.e. central circle and bridge, quite differently [52]. This is also seen in the current study, where there are few visits to the central circle in both adolescence and adulthood, but several visits to the bridge. Thus, the MCSF gives information about different types of risk-assessment and risk-taking behaviours and has been shown to be more sensitive than the open field and elevated plus maze tests [49,56], and it was therefore somewhat surprising that such modest differences were found in these categories. On the other hand, the MCSF test has not been validated for the use in young animals. Explorative strategies and defensive behaviour in areas with potential risks incorporated may differ between young and adult rats [57]. For example, the time spent in the risk assessment areas was quite long for many of the young animals. It is likely that this is not an expression of risk-assessment behaviour, but rather that the slope is primarily considered sheltered by these smaller animals. This possibility will need further investigation in future studies.

The water-drinking rats were used to follow the behavioural development of the three groups subjected to different rearing conditions. In this behavioural profiling the AFR rats served as a control for the behavioural development of a rat that is subjected to conventional animal facility rearing conditions. In the trend analysis in which individual behavioural strategies of relevance to certain functional categories are taken into account, only minor differences between the groups were revealed. In fact, in the second behavioural test the groups were even more similar to each other and with a difference only in shelter seeking when comparing the MS groups relative to AFR. This is in line with previous studies showing few differences in the MSCF test in adult rats that had undergone the MS procedure [58] but contrasts results from repeated testing in adulthood, where a general finding is that rats decrease general activity and risk-taking behaviour [48,56]. It is also possible that the individual housing had a large impact on the animals and that the lack of social interactions was a stressor big enough to make the behaviour become more uniform among the groups [59]. However, in studies using social isolation as a model for schizophrenia it has been pointed out that it is important to start the social isolation during PND21 to 30 in order to have robust effects [60] and in the current study the individual housing began on PND34, after the most sensitive period. The rats were also handled several times a week for weighing and cage changes, clearly deviating from social isolation protocols where handling is kept to a minimum because it attenuates the isolation effects [60]. Effects on behaviour of the single housing would therefore be very hard to attribute only to social isolation. Nevertheless, one of the drawbacks of this study is the lack of group housed controls, but the drinking paradigm made this difficult and nor was this part of the original aim. However, this would be an interesting question for future studies.

Yet another factor that could influence the behaviour in the second MCSF test is the rats’ memory of the arena [48,56]. Brief periods of MS have in many cases been shown to enhance spatial memory performance while results from longer separations are more inconsistent [61]. Even though the time between the two testing occasions was quite long it is likely that the first experience will affect the second testing and that the experience of the first test and/or the memory of this experience are different between the groups.

### Interaction effects of maternal separation and adolescent alcohol drinking

#### Alcohol intake

The intake data from this study confirms previous findings showing that there are no differences in alcohol consumption in rats subjected to different rearing environment when free intermittent access to alcohol is given during adolescence [24]. These results further strengthen the notion of differences in MS-induced effects on alcohol consumption depending on onset of drinking; in adult rats, that are group-housed during adolescence, an increased propensity for high alcohol intake is described [19,53,62]. Adolescent rats have a high alcohol intake and many other factors in the developing brain may be more important than rearing conditions in determining alcohol consumption behaviour [63,64]. However, it also warrants further investigation of individual housing and its effects on adolescent voluntary alcohol consumption. The question of whether these factors, early access and individual housing, are equally important, if they potentiate each other or if one of the factors play a more crucial role is highly interesting. Here, we can conclude that single-housed adolescent rats have the same voluntary alcohol consumption regardless of early life experiences.

#### Behaviour

The results showed that the alcohol-drinking rats did not follow the same behavioural development of general activity as their water-drinking counterparts, independent of MS group. The Wistar rats used are outbred rats and there are large individual variations in behaviour as recently described in evaluations of behavioural profiles in Wistars from different suppliers [65,66]. In addition to differences depending on supplier there are also differences between batches of animals from the same supplier, which is less described in the literature but well acknowledged by researchers. In the present study, it was not possible to create groups from known profiles and the result of the random selection was a more pronounced difference in risk parameters in the water rats compared to the designated alcohol rats already week 4. This is unfortunate, as it affects the interpretation of differences in behavioural development between the intake groups. What is clear is that the MS condition affects the direction of the behavioural development in the risk-taking category as evidenced in the water-drinking rats, with the same pattern in the alcohol rats. In future experiments it would be of interest to further study consequences of alcohol drinking in young rats with a known high or low risk taking profile, but different methods of profiling the young rats should be considered. Although the possibility of studying the effects of alcohol over time in these animals warrants further studies of causality in terms of behaviour and alcohol intake, care should be taken to select animals based on their initial behaviour to make sure to include both ends of the spectra in both intake groups.

#### Relationship between behaviour and alcohol intake

The predictive value of the behavioural profiles in relation to later alcohol intake was weak. One explanation could be that the behavioural profile at 4 weeks of age is not related to their subsequent alcohol intake. It is also possible that other tests will better predict later alcohol intake in young rats or that the higher intake in these young rats could be overshadowing their behavioural profile. The single housing, which the rats were subjected to when the alcohol access began, could also be an important stressor that confounded the effect of the animals’ behavioural profiles on their alcohol intake. For future studies it would be interesting to be able to study the behavioural development in rats with a normal social environment during this period, in combination with alcohol intake.

#### Peptide levels

Levels of ir BEND in the pituitary and the PAG were found to separate the MS15 and MS360. The lower ir levels in MS360 rats may reflect a down-regulated BEND system and the significance of this finding can only be speculated upon. However, it is of interest in light of the differences in hypothalamus-pituitary-adrenal axis function [20,58,62] and in endogenous pain modulating systems due to MS [42]. DYNB and MEAP were not affected by the MS, which is in contrast to previous studies using the MS model, where several differences in ir DYNB and MEAP levels have been found in adulthood between the two MS groups [38-40]. However, in this study the animals were individually housed and given access to alcohol at an early age and throughout adolescence, introducing two factors not previously studied. It was previously reported that the MS-induced effects on opioids are age-dependent [39]. MS360 rats differed from MS15 rats in areas related to stress regulation immediately after MS, whereas in rats that were group-housed through adolescence, differences emerged in reward-related brain areas in adulthood coinciding with the time point when the differences in alcohol consumption appear. These results indicate that the development of opioid networks during adolescence in MS15 and MS360, respectively, contribute to the MS-induced effects seen in adult rats. The present results suggest that individual housing during adolescence attenuate the differences in DYNB and MEAP between group-housed MS15 and MS360 rats which may be a consequence of disturbed social behaviour [43,44].

Alcohol-induced effects on opioid levels after voluntary drinking in adolescence is to our knowledge very scarce, so the increase of DYNB in the hippocampus and MEAP in the amygdala are notable. Early adversity is associated with dysfunction in cognitive and executive functions and recent reviews show that these brain regions are important targets for early-life stress [67,68] and they are implicated in individual differences in addiction processes [69].

Previous studies have shown that dynorphin injections into the hippocampus impair spatial learning in rats and because alcohol also impairs spatial learning it is possible that the two are connected through an alcohol-induced increase of dynorphin [70]. Elevated levels of dynorphin have been found in animals with spatial learning impairments [71] and this kind of impairment has been shown to be diminished in prodynorphin knockout mice [72], further supporting dynorphin’s role in learning and memory acquisition. Contrary to the present study, voluntary alcohol consumption in adulthood had no effects on hippocampal dynorphin either in MS15 or MS360 rats [40]. Differences between that study and the current study are the age of onset of drinking, the duration of drinking and the alcohol intake paradigm. The alcohol-induced up-regulation of dynorphin in the current study could be due to a longer exposure, starting in adolescence. The data is in agreement with a study on human alcoholics with a long history of AUD, where increased levels of dynorphin A and B were found in the hippocampus [73]. In light of previous results of altered behaviour [15,74,75] and brain damage [76] after adolescent alcohol exposure it is interesting that the alcohol-drinking animals do not change their behaviour over time. An alcohol-induced disruption of learning/memory circuits may cause a lack of memory of the first testing occasion.

It is also possible that the intermittent access in the present study contributed to the increase in DYNB not previously seen with continuous alcohol access [40]. Different patterns of for example cocaine administration have different neurobiological effects [77] and binge-like or intermittent patterns of intake have been suggested to be important for development of AUD [78-82]. In future studies, it would be interesting to sort out the contribution of these different parameters, i.e. age of onset, duration of drinking and intake patterns.

The effects on MEAP are in agreement with a previous study where adult alcohol-drinking MS rats had higher ir MEAP levels in the amygdala [40], showing that the amygdala is affected by adolescent as well as adult exposure to alcohol. This result is also consistent with studies investigating mRNA levels of enkephalin or preproenkephalin in adult voluntary drinking rats that show increased mRNA expression in the central nucleus of the amygdala [83,84]. Increased ir MEAP levels in the amygdala could be connected to the anxiolytic effect of alcohol consumption [85-87]. For example, delta-opioid receptor activation in the amygdala has been shown to reduce anxiety-like behaviour [88], although overexpression does not decrease anxiety by itself [89]. Both delta- and mu-opioid receptors are present in the amygdala and there are differences in distribution of the receptors in the different nuclei and subdivisions of the amygdala [90]. Local injections of specific mu- and delta-receptor antagonists in the amygdala decrease self-administration of ethanol, showing that this area is also involved in modulating ethanol consumption [91]. However, a decrease in self-administration is not seen in dependent animals, indicating that the mu- and delta-receptors are mainly involved in the modulation during the acquisition phase and not the dependent state [92]. Because of the variation in enkephalin and receptor distribution it is difficult to draw any definitive conclusions of the functional impact of an increase of ir MEAP levels in the whole of amygdala, such as we dissect it, but the fact that there is an effect on this peptide after voluntary alcohol consumption in both young and adult individuals underlines the importance of further studies of this area in relation to the acquisition of excessive alcohol intake and development of AUD.

Previous studies in adult rats show enhanced alcohol-induced responses on ir MEAP levels in several other brain areas [40]. Such differences in response to alcohol due to rearing conditions were not seen in the present study, showing that early-life environmental conditions have less (or no) influence on alcohol-induced effects on dynorphin or MEAP in the young brain in animals that have been single housed throughout adolescence.

Correlations between behavioural profiles and opioid levels were not considered interesting in this study due to the long interval between the behavioural test and the dissection. Many things affect the opioid levels and even if correlations were found they would be very difficult to interpret.

## Conclusions

The results show that the behavioural development of risk taking is dependent on rearing conditions, but that alcohol-induced effects on general activity are independent of rearing condition. Opioids in the amygdala and hippocampus emerged as interesting targets for adolescent alcohol drinking. Although some methodological modification may have to be considered, the findings show that it is possible to follow behaviour over time using the MCSF, which enables studies of environmental impact, on behaviour. This, in combination with neurobiological investigations, could prove to be useful in elucidating the causal links between early-life stress and adult phenotype in a number of psychiatric diagnoses.

## Supporting Information

Figure S1 **Box plots of the median alcohol preference (%) over time in the alcohol-drinking groups.** Both groups significantly increased their preference over time, Friedman ANOVA, MS15 [χ^2^ = 105; p < 0.001] and MS360 [χ^2^ =111; p < 0.001]. Data are expressed as median, quartile range, min/max and outliers. MS15 = maternal separation 15 min, MS360 = maternal separation 360 min.
(EPS)

Table S1 **Mean ± SEM for each descriptive parameter from the multivariate concentric square field™ (MCSF) test at the two different ages in the different groups.**
(DOCX)

Table S2 **Mean ir beta-endorphin (BEND) levels (fmol/mg tissue) ± SEM in the dissected brain areas in the different groups of rats.**
(DOCX)

Table S3 **Mean ir dynorphin B (DYNB) levels (fmol/mg tissue) ± SEM in the dissected brain areas in the different groups of rats.**
(DOCX)

Table S4 **Mean ir Met-enkephalin-Arg^6^Phe^7^ (MEAP) levels (fmol/mg tissue) ± SEM in the dissected brain areas in the different groups of rats.**
(DOCX)

## References

[1] Gilbert R, Widom CS, Browne K, Fergusson D, Webb E et al. (2009) Burden and consequences of child maltreatment in high-income countries. Lancet 373: 68-81. doi:10.1016/S0140-6736(08)61706-7. PubMed: 19056114. 19056114

[2] Andersen SL, Teicher MH (2009) Desperately driven and no brakes: developmental stress exposure and subsequent risk for substance abuse. Neurosci Biobehav Rev 33: 516-524. doi:10.1016/j.neubiorev.2008.09.009. PubMed: 18938197. 18938197 PMC2688959

[3] McCrory E, De Brito SA, Viding E (2011) The impact of childhood maltreatment: a review of neurobiological and genetic factors. Front Psychiatry 2: 48 PubMed: 21847382. 21847382 10.3389/fpsyt.2011.00048 PMC3148713

[4] Fenton MC, Geier T, Keyes K, Skodol AE, Grant BF et al. (2012) Combined role of childhood maltreatment, family history, and gender in the risk for alcohol dependence. Psychol Med, 43: 1-13. PubMed: 22883538. 22883538 10.1017/S0033291712001729 PMC3767412

[5] Mayfield RD, Harris RA, Schuckit MA (2008) Genetic factors influencing alcohol dependence. Br J Pharmacol 154: 275-287. PubMed: 18362899. 18362899 10.1038/bjp.2008.88 PMC2442454

[6] Langeland W, Draijer N, van den Brink W (2004) Psychiatric comorbidity in treatment-seeking alcoholics: the role of childhood trauma and perceived parental dysfunction. Alcohol Clin Exp Res 28: 441-447. doi:10.1097/01.ALC.0000117831.17383.72. PubMed: 15084902. 15084902

[7] Heilig M, Goldman D, Berrettini W, O’Brien CP (2011) Pharmacogenetic approaches to the treatment of alcohol addiction. Nat Rev Neurosci 12: 670-684. doi:10.1038/nrg3073. PubMed: 22011682. 22011682 PMC3408029

[8] Romeo RD, McEwen BS (2006) Stress and the adolescent brain. Ann N Y Acad Sci 1094: 202-214. doi:10.1196/annals.1376.022. PubMed: 17347352. 17347352

[9] Crews F, He J, Hodge C (2007) Adolescent cortical development: a critical period of vulnerability for addiction. Pharmacol Biochem Behav 86: 189-199. doi:10.1016/j.pbb.2006.12.001. PubMed: 17222895. 17222895 PMC11646682

[10] Cloninger CR, Sigvardsson S, Gilligan SB, von Knorring AL, Reich T et al. (1988) Genetic heterogeneity and the classification of alcoholism. Adv Alcohol Subst Abus 7: 3-16. doi:10.1300/J251v07n03_02. 3066194

[11] Lesch OM, Kefer J, Lentner S, Mader R, Marx B et al. (1990) Diagnosis of chronic alcoholism--classificatory problems. Psychopathology 23: 88-96. doi:10.1159/000284644. PubMed: 2259714. 2259714

[12] Leggio L, Kenna GA, Fenton M, Bonenfant E, Swift RM (2009) Typologies of alcohol dependence. From Jellinek to genetics and beyond. Neuropsychol Rev 19: 115-129. doi:10.1007/s11065-008-9080-z. PubMed: 19184441. 19184441

[13] Alaux-Cantin S, Warnault V, Legastelois R, Botia B, Pierrefiche O et al. (2013) Alcohol intoxications during adolescence increase motivation for alcohol in adult rats and induce neuroadaptations in the nucleus accumbens. Neuropharmacology 67: 521-531. doi:10.1016/j.neuropharm.2012.12.007. PubMed: 23287538. 23287538

[14] Monti PM, Miranda R Jr., Nixon K, Sher KJ, Swartzwelder HS et al. (2005) Adolescence: booze, brains, and behavior. Alcohol Clin Exp Res 29: 207-220. doi:10.1097/01.ALC.0000153551.11000.F3. PubMed: 15714044. 15714044

[15] Nasrallah NA, Yang TW, Bernstein IL (2009) Long-term risk preference and suboptimal decision making following adolescent alcohol use. Proc Natl Acad Sci U S A 106: 17600-17604. doi:10.1073/pnas.0906629106. PubMed: 19805186. 19805186 PMC2765167

[16] Nasrallah NA, Clark JJ, Collins AL, Akers CA, Phillips PE et al. (2011) Risk preference following adolescent alcohol use is associated with corrupted encoding of costs but not rewards by mesolimbic dopamine. Proc Natl Acad Sci U S A 108: 5466-5471. doi:10.1073/pnas.1017732108. PubMed: 21402915. 21402915 PMC3069180

[17] Grota L, Ader R (1969) Continuous recording of maternal behaviour in Rattus Norvegicus. Anim Behav 17: 722-729. doi:10.1016/S0003-3472(69)80019-9. 5530113

[18] Fleming AS, Rosenblatt JS (1974) Maternal behavior in the virgin and lactating rat. J Comp Physiol Psychol 86: 957-972. doi:10.1037/h0036414. 4833599

[19] Roman E, Nylander I (2005) The impact of emotional stress early in life on adult voluntary ethanol intake-results of maternal separation in rats. Stress 8: 157-174. doi:10.1080/10253890500188666. PubMed: 16323264. 16323264

[20] Pryce CR, Feldon J (2003) Long-term neurobehavioral impact of the postnatal environment in rats: manipulations, effects and mediating mechanisms. Neurosci Biobehav Rev 27: 57-71. doi:10.1016/S0149-7634(03)00009-5. PubMed: 12732223. 12732223

[21] Ploj K, Roman E, Nylander I (2003) Long-term effects of maternal separation on ethanol intake and brain opioid and dopamine receptors in male Wistar rats. Neuroscience 121: 787-799. doi:10.1016/S0306-4522(03)00499-8. PubMed: 14568037. 14568037

[22] Jaworski JN, Francis DD, Brommer CL, Morgan ET, Kuhar MJ (2005) Effects of early maternal separation on ethanol intake, GABA receptors and metabolizing enzymes in adult rats. Psychopharmacology (Berl) 181: 8-15. doi:10.1007/s00213-005-2232-4. PubMed: 15830234. 15830234

[23] Huot RL, Thrivikraman KV, Meaney MJ, Plotsky PM (2001) Development of adult ethanol preference and anxiety as a consequence of neonatal maternal separation in Long Evans rats and reversal with antidepressant treatment. Psychopharmacology (Berl) 158: 366-373. doi:10.1007/s002130100701. PubMed: 11797057. 11797057

[24] Daoura L, Haaker J, Nylander I (2011) Early environmental factors differentially affect voluntary ethanol consumption in adolescent and adult male rats. Alcohol Clin Exp Res 35: 506-515. doi:10.1111/j.1530-0277.2010.01367.x. PubMed: 21143247. 21143247

[25] Gustafsson L, Nylander I (2006) Time-dependent alterations in ethanol intake in male wistar rats exposed to short and prolonged daily maternal separation in a 4-bottle free-choice paradigm. Alcohol Clin Exp Res 30: 2008-2016. doi:10.1111/j.1530-0277.2006.00247.x. 17117966

[26] Gianoulakis C (2004) Endogenous opioids and addiction to alcohol and other drugs of abuse. Curr Top Med Chem 4: 39-50. doi:10.2174/1568026043451573. PubMed: 14754375. 14754375

[27] Sanchis-Segura C, Grisel JE, Olive MF, Ghozland S, Koob GF et al. (2005) Role of the endogenous opioid system on the neuropsychopharmacological effects of ethanol: new insights about an old question. Alcohol Clin Exp Res 29: 1522-1527. doi:10.1097/01.alc.0000174913.60384.e8. PubMed: 16156049. 16156049

[28] Drews E, Zimmer A (2010) Modulation of alcohol and nicotine responses through the endogenous opioid system. Prog Neurobiol 90: 1-15. doi:10.1016/j.pneurobio.2009.09.004. PubMed: 19800387. 19800387

[29] Spanagel R (2009) Alcoholism: a systems approach from molecular physiology to addictive behavior. Physiol Rev 89: 649-705. doi:10.1152/physrev.00013.2008. PubMed: 19342616. 19342616

[30] Oswald LM, Wand GS (2004) Opioids and alcoholism. Physiol Behav 81: 339-358. doi:10.1016/j.physbeh.2004.02.008. PubMed: 15159175. 15159175

[31] Nylander I, Silberring J (1998) Opioid peptides in drug dependence and neurological diseases. In: Qureshi GA Parvez H Caudy P Parvez S Neurochemical markers of neurodegenerative nervous diseases and drug addiction. Zeist, The Netherlands: VSP International Science Publisher pp. 171-192.

[32] Gonzales RA, Weiss F (1998) Suppression of ethanol-reinforced behavior by naltrexone is associated with attenuation of the ethanol-induced increase in dialysate dopamine levels in the nucleus accumbens. J Neurosci 18: 10663-10671. PubMed: 9852601. 9852601 10.1523/JNEUROSCI.18-24-10663.1998 PMC6793337

[33] Vengeliene V, Bilbao A, Molander A, Spanagel R (2008) Neuropharmacology of alcohol addiction. Br J Pharmacol 154: 299-315. PubMed: 18311194. 18311194 10.1038/bjp.2008.30 PMC2442440

[34] Volpicelli JR, Alterman AI, Hayashida M, O’Brien CP (1992) Naltrexone in the treatment of alcohol dependence. Arch Gen Psychiatry 49: 876-880. doi:10.1001/archpsyc.1992.01820110040006. PubMed: 1345133. 1345133

[35] O’Malley SS, Jaffe AJ, Chang G, Schottenfeld RS, Meyer RE et al. (1992) Naltrexone and coping skills therapy for alcohol dependence. A controlled study. Arch Gen Psychiatry 49: 881-887. doi:10.1001/archpsyc.1992.01820110045007. PubMed: 1444726. 1444726

[36] Altshuler HL, Phillips PE, Feinhandler DA (1980) Alteration of ethanol self-administration by naltrexone. Life Sci 26: 679-688. doi:10.1016/0024-3205(80)90257-X. PubMed: 6767889. 6767889

[37] Nylander I, Roman E (2012) Neuropeptides as mediators of the early-life impact on the brain; implications for alcohol use disorders. Front. J Mol Neurosci 5: 77. 10.3389/fnmol.2012.00077 PMC3389713 22783165

[38] Ploj K, Roman E, Nylander I (2003) Long-term effects of short and long periods of maternal separation on brain opioid peptide levels in male Wistar rats. Neuropeptides 37: 149-156. doi:10.1016/S0143-4179(03)00043-X. PubMed: 12860112. 12860112

[39] Gustafsson L, Oreland S, Hoffmann P, Nylander I (2008) The impact of postnatal environment on opioid peptides in young and adult male Wistar rats. Neuropeptides 42: 177-191. doi:10.1016/j.npep.2007.10.006. PubMed: 18082882. 18082882

[40] Gustafsson L, Zhou Q, Nylander I (2007) Ethanol-induced effects on opioid peptides in adult male Wistar rats are dependent on early environmental factors. Neuroscience 146: 1137-1149. doi:10.1016/j.neuroscience.2007.02.037. PubMed: 17391858. 17391858

[41] Daoura L, Nylander I (2011) The response to naltrexone in ethanol-drinking rats depends on early environmental experiences. Pharmacol Biochem Behav 99: 626-633. doi:10.1016/j.pbb.2011.06.004. PubMed: 21689677. 21689677

[42] Kalinichev M, Easterling KW, Holtzman SG (2001) Repeated neonatal maternal separation alters morphine-induced antinociception in male rats. Brain. Res Bull 54: 649-654. doi:10.1016/S0361-9230(01)00485-3. 11403991

[43] Panksepp J, Herman BH, Vilberg T, Bishop P, DeEskinazi FG (1980) Endogenous opioids and social behavior. Neurosci Biobehav Rev 4: 473-487. doi:10.1016/0149-7634(80)90036-6. PubMed: 6258111. 6258111

[44] Nelson EE, Panksepp J (1998) Brain substrates of infant-mother attachment: contributions of opioids, oxytocin, and norepinephrine. Neurosci Biobehav Rev 22: 437-452. doi:10.1016/S0149-7634(97)00052-3. PubMed: 9579331. 9579331

[45] Trezza V, Vanderschuren LJ (2008) Cannabinoid and opioid modulation of social play behavior in adolescent rats: differential behavioral mechanisms. Eur Neuropsychopharmacol 18: 519-530. doi:10.1016/S0924-977X(08)70783-1. PubMed: 18434104. 18434104 PMC2490798

[46] Trezza V, Damsteegt R, Achterberg EJ, Vanderschuren LJ (2011) Nucleus accumbens mu-opioid receptors mediate social reward. J Neurosci 31: 6362-6370. doi:10.1523/JNEUROSCI.5492-10.2011. PubMed: 21525276. 21525276 PMC3098965

[47] Vanderschuren LJ, Niesink RJ, Spruijt BM, Van Ree JM (1995) Mu- and kappa-opioid receptor-mediated opioid effects on social play in juvenile rats. Eur J Pharmacol 276: 257-266. doi:10.1016/0014-2999(95)00040-R. PubMed: 7601211. 7601211

[48] Meyerson BJ, Augustsson H, Berg M, Roman E (2006) The Concentric Square Field: a multivariate test arena for analysis of explorative strategies. Behav Brain Res 168: 100-113. doi:10.1016/j.bbr.2005.10.020. 16356558

[49] Roman E, Stewart RB, Bertholomey ML, Jensen ML, Colombo G et al. (2012) Behavioral profiling of multiple pairs of rats selectively bred for high and low alcohol intake using the MCSF test. Addict Biol 17: 33-46. doi:10.1111/j.1369-1600.2011.00327.x. PubMed: 21521426. 21521426 PMC5472351

[50] Christensson-Nylander I, Nyberg F, Ragnarsson U, Terenius L (1985) A general procedure for analysis of proenkephalin B derived opioid peptides. Regul Pept 11: 65-76. doi:10.1016/0167-0115(85)90032-1. PubMed: 2861627. 2861627

[51] Nylander I, Stenfors C, Tan-No K, Mathé AA, Terenius L (1997) A comparison between microwave irradiation and decapitation: basal levels of dynorphin and enkephalin and the effect of chronic morphine treatment on dynorphin peptides. Neuropeptides 31: 357-365. doi:10.1016/S0143-4179(97)90072-X. PubMed: 9308024. 9308024

[52] Meyerson BJ, Jurek B, Roman E (2013) A Rank-Order Procedure Applied to an Ethoexperimental Behavior Model - The Multivariate Concentric Square Field ™ (MCSF) Test. Behav Brain Sci (In press).

[53] Moffett MC, Vicentic A, Kozel M, Plotsky P, Francis DD et al. (2007) Maternal separation alters drug intake patterns in adulthood in rats. Biochem Pharmacol 73: 321-330. doi:10.1016/j.bcp.2006.08.003. PubMed: 16962564. 16962564 PMC2692348

[54] Farkas J, Reglodi D, Gaszner B, Szogyi D, Horvath G et al. (2009) Effects of maternal separation on the neurobehavioral development of newborn Wistar rats. Brain. Res Bull 79: 208-214. doi:10.1016/j.brainresbull.2008.12.011. 19150489

[55] Ploj K, Roman E, Nylander I (2002) Effects of maternal separation on brain nociceptin/orphanin FQ peptide levels in male Wistar rats. Pharmacol Biochem Behav 73: 123-129. doi:10.1016/S0091-3057(02)00778-5. PubMed: 12076731. 12076731

[56] Roman E, Meyerson BJ, Hyytia P, Nylander I (2007) The multivariate concentric square field test reveals different behavioural profiles in male AA and ANA rats with regard to risk taking and environmental reactivity. Behav Brain Res 183: 195-205. doi:10.1016/j.bbr.2007.06.009. PubMed: 17688955. 17688955

[57] Wiedenmayer CP (2009) Plasticity of defensive behavior and fear in early development. Neurosci Biobehav Rev 33: 432-441. doi:10.1016/j.neubiorev.2008.11.004. PubMed: 19073211. 19073211 PMC2671008

[58] Roman E, Gustafsson L, Berg M, Nylander I (2006) Behavioral profiles and stress-induced corticosteroid secretion in male Wistar rats subjected to short and prolonged periods of maternal separation. Horm Behav 50: 736-747. doi:10.1016/j.yhbeh.2006.06.016. PubMed: 16876800. 16876800

[59] Trezza V, Baarendse PJ, Vanderschuren LJ (2010) The pleasures of play: pharmacological insights into social reward mechanisms. Trends Pharmacol Sci 31: 463-469. doi:10.1016/j.tips.2010.06.008. PubMed: 20684996. 20684996 PMC2946511

[60] Fone KC, Porkess MV (2008) Behavioural and neurochemical effects of post-weaning social isolation in rodents-relevance to developmental neuropsychiatric disorders. Neurosci Biobehav Rev 32: 1087-1102. doi:10.1016/j.neubiorev.2008.03.003. PubMed: 18423591. 18423591

[61] Kosten TA, Kim JJ, Lee HJ (2012) Early life manipulations alter learning and memory in rats. Neurosci Biobehav Rev 36: 1985-2006. doi:10.1016/j.neubiorev.2012.07.003. PubMed: 22819985. 22819985 PMC3463710

[62] Becker HC, Lopez MF, Doremus-Fitzwater TL (2011) Effects of stress on alcohol drinking: a review of animal studies. Psychopharmacology (Berl) 218: 131-156. doi:10.1007/s00213-011-2443-9. PubMed: 21850445. 21850445 PMC3247761

[63] Vetter CS, Doremus-Fitzwater TL, Spear LP (2007) Time course of elevated ethanol intake in adolescent relative to adult rats under continuous, voluntary-access conditions. Alcohol Clin Exp Res 31: 1159-1168. doi:10.1111/j.1530-0277.2007.00417.x. PubMed: 17511750. 17511750 PMC2094127

[64] García-Burgos D, González F, Manrique T, Gallo M (2009) Patterns of ethanol intake in preadolescent, adolescent, and adult Wistar rats under acquisition, maintenance and relapse-like conditions. Alcohol Clin Exp Res 33: 722-728. doi:10.1111/j.1530-0277.2008.00889.x. PubMed: 19183130. 19183130

[65] Palm S, Hävermark Å, Meyerson BJ, Nylander I, Roman E (2011) When is a Wistar a Wistar? Behavioral profiling of outbred Wistar rats from five different suppliers using the MCSF test. Appl Anim Behav Sci 135: 128-137. doi:10.1016/j.applanim.2011.08.010.

[66] Goepfrich AA, Gluch C, Friemel CM, Schneider M (2013) Behavioral differences in three Wistar Han rat lines for emotional reactivity, cognitive processing and ethanol intake. Physiol Behav 110-111: 102-108. doi:10.1016/j.physbeh.2012.12.019. PubMed: 23306104. 23306104

[67] Pechtel P, Pizzagalli DA (2011) Effects of early life stress on cognitive and affective function: an integrated review of human literature. Psychopharmacology (Berl) 214: 55-70. doi:10.1007/s00213-010-2009-2. PubMed: 20865251. 20865251 PMC3050094

[68] Tottenham N, Sheridan MA (2009) A review of adversity, the amygdala and the hippocampus: a consideration of developmental timing. Front Hum Neurosci 3: 68 PubMed: 20161700. 20161700 10.3389/neuro.09.068.2009 PMC2813726

[69] Koob GF, Volkow ND (2010) Neurocircuitry of addiction. Neuropsychopharmacology 35: 217-238. doi:10.1038/npp.2009.110. PubMed: 19710631. 19710631 PMC2805560

[70] Sandin J, Nylander I, Georgieva J, Schött PA, Ogren SO et al. (1998) Hippocampal dynorphin B injections impair spatial learning in rats: a kappa-opioid receptor-mediated effect. Neuroscience 85: 375-382. doi:10.1016/S0306-4522(97)00605-2. PubMed: 9622237. 9622237

[71] Jiang HK, Owyang VV, Hong JS, Gallagher M (1989) Elevated dynorphin in the hippocampal formation of aged rats: relation to cognitive impairment on a spatial learning task. Proc Natl Acad Sci U S A 86: 2948-2951. doi:10.1073/pnas.86.8.2948. PubMed: 2565040. 2565040 PMC287037

[72] Nguyen XV, Masse J, Kumar A, Vijitruth R, Kulik C et al. (2005) Prodynorphin knockout mice demonstrate diminished age-associated impairment in spatial water maze performance. Behav Brain Res 161: 254-262. doi:10.1016/j.bbr.2005.02.010. PubMed: 15922052. 15922052

[73] Bazov I, Kononenko O, Watanabe H, Kuntić V, Sarkisyan D et al. (2013) The endogenous opioid system in human alcoholics: molecular adaptations in brain areas involved in cognitive control of addiction. Addict Biol 18: 161-169. doi:10.1111/j.1369-1600.2011.00366.x. PubMed: 21955155. 21955155

[74] Clark JJ, Nasrallah NA, Hart AS, Collins AL, Bernstein IL et al. (2012) Altered risk-based decision making following adolescent alcohol use results from an imbalance in reinforcement learning in rats. PLOS ONE 7: e37357. doi:10.1371/journal.pone.0037357. PubMed: 22615989. 22615989 PMC3353889

[75] White AM, Ghia AJ, Levin ED, Swartzwelder HS (2000) Binge pattern ethanol exposure in adolescent and adult rats: differential impact on subsequent responsiveness to ethanol. Alcohol Clin Exp Res 24: 1251-1256. doi:10.1111/j.1530-0277.2000.tb02091.x. PubMed: 10968665. 10968665

[76] Crews FT, Braun CJ, Hoplight B, Switzer RC 3rd, Knapp DJ (2000) Binge ethanol consumption causes differential brain damage in young adolescent rats compared with adult rats. Alcohol Clin Exp Res 24: 1712-1723. doi:10.1111/j.1530-0277.2000.tb01973.x. PubMed: 11104119. 11104119

[77] Unterwald EM, Kreek MJ, Cuntapay M (2001) The frequency of cocaine administration impacts cocaine-induced receptor alterations. Brain Res 900: 103-109. doi:10.1016/S0006-8993(01)02269-7. PubMed: 11325352. 11325352

[78] Koob G, Kreek MJ (2007) Stress, dysregulation of drug reward pathways, and the transition to drug dependence. Am J Psychiatry 164: 1149-1159. doi:10.1176/appi.ajp.2007.05030503. PubMed: 17671276. 17671276 PMC2837343

[79] Sprow GM, Thiele TE (2012) The neurobiology of binge-like ethanol drinking: evidence from rodent models. Physiol Behav 106: 325-331. doi:10.1016/j.physbeh.2011.12.026. PubMed: 22245775. 22245775 PMC3348336

[80] Hopf FW, Chang SJ, Sparta DR, Bowers MS, Bonci A (2010) Motivation for alcohol becomes resistant to quinine adulteration after 3 to 4 months of intermittent alcohol self-administration. Alcohol Clin Exp Res 34: 1565-1573. doi:10.1111/j.1530-0277.2010.01241.x. PubMed: 20586757. 20586757 PMC2997761

[81] Lesscher HM, van Kerkhof LW, Vanderschuren LJ (2010) Inflexible and indifferent alcohol drinking in male mice. Alcohol Clin Exp Res 34: 1219-1225. PubMed: 20477770. 20477770 10.1111/j.1530-0277.2010.01199.x

[82] Seif T, Chang SJ, Simms JA, Gibb SL, Dadgar J et al. (2013) Cortical activation of accumbens hyperpolarization-active NMDARs mediates aversion-resistant alcohol intake. Nat Neurosci 16: 1094-1100. doi:10.1038/nn.3445. PubMed: 23817545. 23817545 PMC3939030

[83] Cowen MS, Lawrence AJ (2001) Alterations in central preproenkephalin mRNA expression after chronic free-choice ethanol consumption by Fawn-Hooded rats. Alcohol Clin Exp Res 25: 1126-1133. doi:10.1111/j.1530-0277.2001.tb02326.x. PubMed: 11505043. 11505043

[84] Chang GQ, Barson JR, Karatayev O, Chang SY, Chen YW et al. (2010) Effect of chronic ethanol on enkephalin in the hypothalamus and extra-hypothalamic areas. Alcohol Clin Exp Res 34: 761-770. doi:10.1111/j.1530-0277.2010.01148.x. PubMed: 20184566. 20184566 PMC4467542

[85] Wilson MA, Burghardt PR, Lugo JN Jr., Primeaux SD, Wilson SP (2003) Effect of amygdalar opioids on the anxiolytic properties of ethanol. Ann N Y Acad Sci 985: 472-475. PubMed: 12724179. 12724179 10.1111/j.1749-6632.2003.tb07102.x

[86] Bilkei-Gorzo A, Racz I, Michel K, Zimmer A, Klingmüller D et al. (2004) Behavioral phenotype of pre-proenkephalin-deficient mice on diverse congenic backgrounds. Psychopharmacology (Berl) 176: 343-352. doi:10.1007/s00213-004-1904-9. PubMed: 15197532. 15197532

[87] König M, Zimmer AM, Steiner H, Holmes PV, Crawley JN et al. (1996) Pain responses, anxiety and aggression in mice deficient in pre-proenkephalin. Nature 383: 535-538. doi:10.1038/383535a0. PubMed: 8849726. 8849726

[88] Randall-Thompson JF, Pescatore KA, Unterwald EM (2010) A role for delta opioid receptors in the central nucleus of the amygdala in anxiety-like behaviors. Psychopharmacology (Berl) 212: 585-595. doi:10.1007/s00213-010-1980-y. PubMed: 20730419. 20730419 PMC3990196

[89] Kang W, Wilson SP, Wilson MA (2000) Overexpression of proenkephalin in the amygdala potentiates the anxiolytic effects of benzodiazepines. Neuropsychopharmacology 22: 77-88. doi:10.1016/S0893-133X(99)00090-1. PubMed: 10633493. 10633493

[90] Poulin JF, Chevalier B, Laforest S, Drolet G (2006) Enkephalinergic afferents of the centromedial amygdala in the rat. J Comp Neurol 496: 859-876. doi:10.1002/cne.20956. PubMed: 16628615. 16628615

[91] Hyytiä P, Kiianmaa K (2001) Suppression of ethanol responding by centrally administered CTOP and naltrindole in AA and Wistar rats. Alcohol Clin Exp Res 25: 25-33. doi:10.1111/j.1530-0277.2001.tb02123.x. PubMed: 11198711. 11198711

[92] Kissler JL, Sirohi S, Reis DJ, Jansen HT, Quock RM et al. (2013) The One-Two Punch of Alcoholism: Role of Central Amygdala Dynorphins/Kappa-Opioid Receptors. Biol Psychiatry. PubMed: 23611261 10.1016/j.biopsych.2013.03.014 PMC3749293 23611261

