# Supplementary material for: Effects of Rearing Conditions on Behaviour and Endogenous Opioids in Rats with Alcohol Access during Adolescence
Source: PLoS One. 2013 Oct 2;8(10):e76591. doi: 10.1371/journal.pone.0076591 (PMC3788749; doi:10.1371/journal.pone.0076591)
Supplement: Table S1 — Mean ± SEM for each descriptive parameter from the multivariate concentric square field™ (MCSF) test at the two different ages in the different groups. (DOCX) [file pone.0076591.s002.docx]

**Table S1.** Mean ± SEM for each descriptive parameter from the multivariate concentric square field™ (MCSF) test at the two different ages in the different groups.

| **Functional categories** | **Parameters** | **Age (w)** | **AFR** | | | **MS15W** | | | **MS360W** | | | **MS15E** | | | **MS360E** | | | **Interaction effects** |
| --- | --- | --- | --- | --- | --- | --- | --- | --- | --- | --- | --- | --- | --- | --- | --- | --- | --- | --- |
| General  activity | TOTACT | 4 | 73.1 | ± | 3.9 | 41.0 | ± | 13° | 79.7 | ± | 14*^&^ | 46.0 | ± | 5.9^§^ | 59.8 | ± | 7.1^&@^ | Age x MS group [F(1, 56)=9.36; p=0.003] |
|  |  | 15 | 78.4 | ± | 11 | 66.3 | ± | 8.8^+^ | 74.1 | ± | 7.0 | 76.8 | ± | 4.8^+^ | 79.1 | ± | 3.1^+^ |  |
|  | F TOTCORR | 4 | 24.5 | ± | 1.1 | 13.7 | ± | 4.3° | 21.7 | ± | 5.1*^&^ | 13.1 | ± | 1.8 | 18.8 | ± | 2.8^&@^ | Age x MS group [F(1, 56)=4.85; p=0.03] |
|  |  | 15 | 29.4 | ± | 4.2 | 25.0 | ± | 3.7^+^ | 26.5 | ± | 2.6 | 28.6 | ± | 1.8^+^ | 30.5 | ± | 1.4^+^ |  |
|  | F CENTER | 4 | 9.9 | ± | 1.0 | 4.9 | ± | 1.6 | 8.4 | ± | 2.2 | 4.2 | ± | 0.7 | 5.8 | ± | 1.1 | Age x Intake group  [F(1, 56)=4.02; p=0.049] |
|  |  | 15 | 11.7 | ± | 2.3 | 8.1 | ± | 1.8^+^ | 9.8 | ± | 1.7 | 10.5 | ± | 1.1^+^ | 10.9 | ± | 0.9^+^ |  |
|  | D CENTER | 4 | 190 | ± | 38 | 331 | ± | 146 | 123 | ± | 15 | 165 | ± | 57 | 119 | ± | 29 |  |
|  |  | 15 | 92.5 | ± | 15^+^ | 120 | ± | 6.4 | 123 | ± | 14 | 130 | ± | 9.3 | 127 | ± | 12 |  |
|  | D/F CENTER | 4 | 18.8 | ± | 3.1 | 227 | ± | 124° | 40.0 | ± | 17*^&^ | 77.2 | ± | 34 | 32.0 | ± | 10^&^ |  |
|  |  | 15 | 8.8 | ± | 1.3 | 24.2 | ± | 5.2^+^ | 14.3 | ± | 2.4 | 13.9 | ± | 1.1^+^ | 11.9 | ± | 0.7^+^ |  |
| Exploratory  activity | L LEAVE | 4 | 81.2 | ± | 34 | 70.1 | ± | 32 | 68.5 | ± | 15 | 69.6 | ± | 22 | 74.5 | ± | 30 |  |
|  |  | 15 | 18.2 | ± | 2.2 | 45.3 | ± | 11 | 29.2 | ± | 8.0 | 37.7 | ± | 4.3 | 25.0 | ± | 2.4 |  |
|  | OCC LEAVE | 4 | 10/10 | | | 8/10 | | | 10/10 | | | 19/20 | | | 20/20 | | |  |
|  |  | 15 | 9/9 | | | 10/10 | | | 10/10 | | | 20/20 | | | 20/20 | | |  |
|  | D TOTCORR | 4 | 377 | ± | 23 | 304 | ± | 101 | 277 | ± | 46 | 282 | ± | 36 | 305 | ± | 22 |  |
|  |  | 15 | 422 | ± | 20 | 438 | ± | 26^+^ | 502 | ± | 30 | 484 | ± | 21^+^ | 484 | ± | 22^+^ |  |
|  | D/F TOTCORR | 4 | 15.7 | ± | 1.2 | 51.8 | ± | 36 | 16.5 | ± | 1.9 | 22.7 | ± | 2.1 | 19.9 | ± | 1.6 |  |
|  |  | 15 | 16.0 | ± | 1.7 | 21.5 | ± | 3.3 | 20.1 | ± | 1.8 | 18.0 | ± | 1.2 | 16.2 | ± | 0.8 |  |
|  | L HURDLE | 4 | 195 | ± | 64 | 80.1 | ± | 34 | 293 | ± | 62 | 363 | ± | 39 | 444 | ± | 82 | Age x Intake group [F(1, 44)=11.4; p=0.002] |
|  |  | 15 | 146 | ± | 49 | 167 | ± | 16 | 292 | ± | 66 | 133 | ± | 25 | 127 | ± | 21 |  |
|  | OCC HURDLE | 4 | 10/10 | | | 4/10° | | | 8/10 | | | 17/20 | | | 19/20 | | |  |
|  |  | 15 | 9/9 | | | 10/10^+^ | | | 10/10 | | | 20/20 | | | 20/20 | | |  |
|  | F HURDLE | 4 | 5.8 | ± | 0.6 | 3.0 | ± | 1.2° | 4.8 | ± | 1.4 | 3.0 | ± | 0.5 | 4.4 | ± | 0.8 |  |
|  |  | 15 | 7.7 | ± | 1.5 | 8.0 | ± | 1.0^+^ | 9.2 | ± | 1.2^+^ | 9.3 | ± | 0.6^+^ | 9.6 | ± | 0.6^+^ |  |
|  | D HURDLE | 4 | 103 | ± | 6.1 | 52.4 | ± | 23° | 70.8 | ± | 17*^&^ | 151 | ± | 54^&^ | 94.6 | ± | 16^&^ | Age x Intake group  [F(1, 56)=5.09; p=0.03] |
|  |  | 15 | 110 | ± | 13 | 111 | ± | 8.6^+^ | 118 | ± | 13^+^ | 131 | ± | 11 | 144 | ± | 9.1^+^ |  |
|  | D/F HURDLE | 4 | 18.8 | ± | 1.3 | 7.4 | ± | 3.5° | 14.9 | ± | 3.4*^&^ | 86.9 | ± | 56^&§^ | 40.8 | ± | 12^&^ | Age x Intake group [F(1, 56)=12.7; p<0.001] |
|  |  | 15 | 17.5 | ± | 2.9 | 15.2 | ± | 1.5^+^ | 13.5 | ± | 1.2 | 14.3 | ± | 0.8 | 16.3 | ± | 1.7^+^ |  |
|  | PC COUNTS | 4 | 0.0 | ± | 0.0 | 0.1 | ± | 0.1 | 0.4 | ± | 0.4 | 0.7 | ± | 0.4 | 1.0 | ± | 0.5 |  |
|  |  | 15 | 7.2 | ± | 2.7^+^ | 4.3 | ± | 1.7^+^ | 6.7 | ± | 2.0^+^ | 5.4 | ± | 1.3^+^ | 7.4 | ± | 1.1^+^ |  |
|  | REARING | 4 | 37.8 | ± | 3.7 | 12.6 | ± | 3.6° | 23.2 | ± | 4.2°*^&^ | 16.5 | ± | 1.4^&^ | 18.3 | ± | 2.0^&^ |  |
|  |  | 15 | 29.8 | ± | 4.5 | 32.4 | ± | 3.3^+^ | 36.8 | ± | 2.4^+^ | 34.4 | ± | 2.7^+^ | 34.6 | ± | 2.2^+^ |  |
| Risk  assessment | L SLOPE | 4 | 241 | ± | 73 | 182 | ± | 32 | 134 | ± | 26 | 193 | ± | 50 | 202 | ± | 46 |  |
|  |  | 15 | 159 | ± | 48 | 147 | ± | 23 | 282 | ± | 81 | 134 | ± | 24 | 127 | ± | 23 |  |
|  | OCC SLOPE | 4 | 10/10 | | | 5/10° | | | 10/10* | | | 17/20 | | | 18/20 | | |  |
|  |  | 15 | 9/9 | | | 10/10^+^ | | | 10/10 | | | 20/20 | | | 20/20 | | |  |
|  | F SLOPE | 4 | 12.0 | ± | 1.0 | 6.2 | ± | 2.3° | 15.8 | ± | 2.0*^&^ | 9.6 | ± | 1.3^&§^ | 11.3 | ± | 1.2^&^ | MS group x Intake group [F(1, 56)=5.62; p=0.02]; Age x MS group [F(1, 56)=11.7; p=0.001]; Age x MS group x Intake group [F(1, 56)=5.61; p=0.02] |
|  |  | 15 | 9.0 | ± | 1.2 | 8.8 | ± | 0.8^+^ | 9.5 | ± | 1.6^+^ | 10.2 | ± | 0.5 | 9.7 | ± | 0.6 |  |
|  | D SLOPE | 4 | 129 | ± | 20 | 88.2 | ± | 31° | 287 | ± | 73*^&^ | 227 | ± | 45^&^ | 265 | ± | 60^&^ | MS group x Intake group [F(1, 56)=4.43; p=0.04]; Age x MS group [F(1, 56)=10.8; p=0.002]; Age x MS group x Intake group [F(1, 56)=6.54; p=0.01] |
|  |  | 15 | 123 | ± | 32 | 137 | ± | 37^+^ | 99.0 | ± | 11 | 162 | ± | 41 | 129 | ± | 32 |  |
|  | D/F SLOPE | 4 | 10.6 | ± | 1.5 | 9.7 | ± | 5.1° | 28.3 | ± | 14*^&^ | 24.3 | ± | 6.8^&^ | 26.4 | ± | 8.2^&^ | Age x MS group [F(1, 56)=7.15; p=0.01]; Age x MS group x Intake group [F(1, 56)=5.32; p=0.03] |
|  |  | 15 | 14.0 | ± | 2.5 | 15.8 | ± | 3.4^+^ | 12.6 | ± | 2.5 | 17.0 | ± | 4.9 | 12.6 | ± | 2.4 |  |
|  | L BE | 4 | 269 | ± | 70 | 218 | ± | 32 | 183 | ± | 44 | 285 | ± | 70 | 266 | ± | 54 |  |
|  |  | 15 | 166 | ± | 48 | 212 | ± | 43 | 315 | ± | 87 | 170 | ± | 24 | 205 | ± | 42 |  |
|  | OCC BE | 4 | 10/10 | | | 5/10° | | | 10/10* | | | 16/20 | | | 18/20 | | |  |
|  |  | 15 | 9/9 | | | 10/10^+^ | | | 10/10 | | | 20/20 | | | 20/20 | | |  |
|  | F BE | 4 | 10.5 | ± | 1.0 | 5.8 | ± | 2.1° | 16.6 | ± | 2.3*^&^ | 9.1 | ± | 1.4^&§^ | 10.3 | ± | 1.3^&^ | MS group x Intake group [F(1, 56)=5.24; p=0.03]; Age x MS group [F(1, 56)=8.02; p=0.006] |
|  |  | 15 | 8.1 | ± | 1.2 | 7.1 | ± | 0.9^+^ | 8.7 | ± | 1.3^+^ | 8.1 | ± | 0.6 | 8.0 | ± | 0.6 |  |
|  | D BE | 4 | 41.6 | ± | 6.2 | 26.9 | ± | 9.6° | 76.7 | ± | 13*^&^ | 52.2 | ± | 8.5^&§^ | 55.9 | ± | 10^&^ | MS group x Intake group [F(1, 56)=5.18; p=0.03]; Age x MS group [F(1, 56)=8.06; p=0.006] |
|  |  | 15 | 27.4 | ± | 4.3 | 33.0 | ± | 8.2^+^ | 38.2 | ± | 9.5 | 31.5 | ± | 3.1 | 29.5 | ± | 3.5 |  |
|  | D/F BE | 4 | 4.0 | ± | 0.4 | 2.6 | ± | 1.0° | 5.5 | ± | 1.0*^&^ | 5.0 | ± | 0.8^&^ | 5.3 | ± | 0.7^&^ | Age x MS group [F(1, 56)=5.91; p=0.02] |
|  |  | 15 | 3.5 | ± | 0.4 | 4.7 | ± | 0.8^+^ | 4.2 | ± | 0.8 | 3.9 | ± | 0.3 | 3.6 | ± | 0.3 |  |
|  | SAP TO CENTER | 4 | 1.4 | ± | 0.6 | 2.0 | ± | 0.8 | 2.1 | ± | 0.6 | 1.5 | ± | 0.3 | 1.8 | ± | 0.2 |  |
|  |  | 15 | 1.9 | ± | 0.5 | 1.8 | ± | 0.4 | 3.0 | ± | 0.5 | 2.5 | ± | 0.4 | 2.8 | ± | 0.4 |  |
|  | OCC SAP CENTER | 4 | 5/10 | | | 6/10 | | | 8/10 | | | 16/20 | | | 19/20 | | |  |
|  |  | 15 | 8/9 | | | 9/10 | | | 8/10 | | | 18/20 | | | 17/20 | | |  |
| Risk taking | L BRIDGE | 4 | 312 | ± | 78 | 247 | ± | 27 | 195 | ± | 44 | 319 | ± | 57 | 316 | ± | 58 |  |
|  |  | 15 | 168 | ± | 47 | 217 | ± | 43 | 318 | ± | 87 | 181 | ± | 24 | 210 | ± | 42 |  |
|  | OCC BRIDGE | 4 | 10/10 | | | 5/10° | | | 10/10* | | | 15/20 | | | 16/20 | | |  |
|  |  | 15 | 9/9 | | | 10/10^+^ | | | 10/10 | | | 20/20 | | | 20/20 | | |  |
|  | F BRIDGE | 4 | 4.9 | ± | 0.5 | 2.8 | ± | 1.0° | 8.0 | ± | 1.1*^&^ | 4.5 | ± | 0.8^§^ | 5.3 | ± | 0.8^&§^ | MS group x Intake group [F(1, 56)=4.59; p=0.04]; Age x MS group [F(1, 56)=6.65; p=0.01] |
|  |  | 15 | 4.0 | ± | 0.6 | 3.6 | ± | 0.4^+^ | 4.3 | ± | 0.6^+^ | 4.0 | ± | 0.3 | 3.9 | ± | 0.3 |  |
|  | D BRIDGE | 4 | 229 | ± | 33 | 168 | ± | 67° | 320 | ± | 51*^&^ | 224 | ± | 46^§^ | 212 | ± | 33^&^ | Age x MS group [F(1, 56)=6.46; p=0.01]; Age x MS group x Intake group [F(1, 56)=4.30; p=0.04] |
|  |  | 15 | 123 | ± | 9.9 | 200 | ± | 40^+^ | 147 | ± | 23 | 132 | ± | 12 | 131 | ± | 14 |  |
|  | D/F BRIDGE | 4 | 47.3 | ± | 5.0 | 38.2 | ± | 21° | 47.1 | ± | 8.2*^&^ | 40.4 | ± | 8.6 | 35.7 | ± | 6.3^&^ | Age x MS group [F(1, 56)=5.58; p=0.02]; Age x MS group x Intake group [F(1, 56)=4.33; p=0.04] |
|  |  | 15 | 35.2 | ± | 4.8 | 71.7 | ± | 24^+^ | 35.3 | ± | 3.6 | 34.8 | ± | 2.7 | 32.9 | ± | 2.3 |  |
|  | L CTRCI | 4 | 352 | ± | 167 | 62.1 | ± | 43 | 256 | ± | 133 | 363 | ± | 137 | 601 | ± | 127 |  |
|  |  | 15 | 567 | ± | 141 | 435 | ± | 130 | 597 | ± | 200 | 421 | ± | 110 | 530 | ± | 94 |  |
|  | OCC CTRCI | 4 | 5/10 | | | 3/10 | | | 5/10 | | | 6/20 | | | 9/20 | | |  |
|  |  | 15 | 6/9 | | | 5/10 | | | 7/10 | | | 14/20^+^ | | | 13/20 | | |  |
|  | F CTRCI | 4 | 1.3 | ± | 0.5 | 0.6 | ± | 0.4 | 1.3 | ± | 0.5 | 0.4 | ± | 0.2 | 0.6 | ± | 0.2 |  |
|  |  | 15 | 1.1 | ± | 0.4 | 0.8 | ± | 0.3 | 1.5 | ± | 0.6 | 1.5 | ± | 0.4^+^ | 0.8 | ± | 0.2 |  |
|  | D CTRCI | 4 | 3.2 | ± | 1.6 | 1.0 | ± | 0.7 | 1.9 | ± | 0.7 | 1.2 | ± | 0.6 | 1.3 | ± | 0.5 |  |
|  |  | 15 | 3.2 | ± | 1.5 | 2.5 | ± | 1.3 | 3.5 | ± | 1.2 | 3.9 | ± | 1.0^+^ | 1.5 | ± | 0.3 |  |
|  | D/F CTRCI | 4 | 1.2 | ± | 0.7 | 0.5 | ± | 0.2 | 0.8 | ± | 0.3 | 1.0 | ± | 0.5 | 0.9 | ± | 0.3 |  |
|  |  | 15 | 2.3 | ± | 1.3 | 2.0 | ± | 1.3 | 2.1 | ± | 0.9 | 2.0 | ± | 0.4^+^ | 1.9 | ± | 0.2 |  |
| Shelter  seeking | L DCR | 4 | 384 | ± | 58 | 416 | ± | 184 | 504 | ± | 192 | 342 | ± | 104 | 272 | ± | 81 |  |
|  |  | 15 | 128 | ± | 49 | 321 | ± | 130 | 356 | ± | 134 | 313 | ± | 64 | 263 | ± | 66 |  |
|  | OCC DCR | 4 | 10/10 | | | 6/10° | | | 6/10° | | | 10/20 | | | 11/20 | | |  |
|  |  | 15 | 9/9 | | | 8/10 | | | 10/10^+^ | | | 18/20^+^ | | | 19/20^+^ | | |  |
|  | F DCR | 4 | 4.2 | ± | 0.4 | 4.0 | ± | 1.2 | 3.1 | ± | 1.1 | 2.2 | ± | 0.6 | 3.6 | ± | 0.9 |  |
|  |  | 15 | 7.4 | ± | 1.0 | 4.9 | ± | 1.2 | 4.6 | ± | 0.8^+^ | 4.8 | ± | 0.7^+^ | 5.8 | ± | 0.5^+^ |  |
|  | D DCR | 4 | 128 | ± | 17 | 229 | ± | 112 | 46.2 | ± | 16 | 98.8 | ± | 37 | 124 | ± | 42 |  |
|  |  | 15 | 300 | ± | 47 | 159 | ± | 47 | 170 | ± | 49^+^ | 126 | ± | 27^+^ | 156 | ± | 21^+^ |  |
|  | D/F DCR | 4 | 30.8 | ± | 2.9 | 31.3 | ± | 15 | 9.2 | ± | 2.6° | 25.1 | ± | 11 | 20.9 | ± | 6.6 |  |
|  |  | 15 | 48.1 | ± | 10 | 25.3 | ± | 6.0 | 31.4 | ± | 7.5^+^ | 23.0 | ± | 5.6^+^ | 25.1 | ± | 2.7^+^ |  |
| Other | GROOMING | 4 | 1.2 | ± | 0.3 | 1.7 | ± | 0.7 | 0.6 | ± | 0.2 | 1.2 | ± | 0.2 | 1.3 | ± | 0.2 |  |
|  |  | 15 | 0.3 | ± | 0.2 | 1.1 | ± | 0.4 | 0.7 | ± | 0.3 | 1.2 | ± | 0.2 | 0.7 | ± | 0.2 |  |
|  | BOLI | 4 | 0.9 | ± | 0.5 | 1.5 | ± | 0.7 | 0.8 | ± | 0.3 | 1.2 | ± | 0.4 | 0.3 | ± | 0.1 |  |
|  |  | 15 | 2.3 | ± | 0.5 | 2.7 | ± | 0.6 | 1.5 | ± | 0.4 | 3.1 | ± | 0.4 | 2.7 | ± | 0.5 |  |
|  | OCC BOLI | 4 | 4/10 | | | 4/10 | | | 5/10 | | | 10/20 | | | 5/20 | | |  |
|  |  | 15 | 9/9^+^ | | | 9/10^+^ | | | 5/10° | | | 18/20^+^ | | | 14/20^+^ | | |  |
|  | URINE | 4 | 0.4 | ± | 0.2 | 0.8 | ± | 0.2 | 0.3 | ± | 0.2 | 0.6 | ± | 0.2 | 0.5 | ± | 0.2 | MS group x Intake group [F(1, 56)=5.69; p=0.02] |
|  |  | 15 | 0.0 | ± | 0.0 | 0.8 | ± | 0.4 | 0.1 | ± | 0.1 | 0.1 | ± | 0.1 | 0.3 | ± | 0.2 |  |
|  | OCC URINE | 4 | 4/10 | | | 7/10 | | | 3/10 | | | 9/20 | | | 7/20 | | |  |
|  |  | 15 | 0/9 | | | 4/10° | | | 3/10 | | | 1/20^+^ | | | 3/20 | | |  |

Behavioural parameters recorded during the 20-min trial of the MCSF test. Occurrence (OCC) is shown for the latency measure for the zones that were not visited by all animals in each group, latencies were treated as missing values if the zones were not visited. It is important to note that after the first behavioural test the rats were randomly assigned to an intake group. This resulted in a difference in a few behavioural parameters between alcohol and water groups before the intake had begun.

*Abbreviations:* BE = bridge entrance; CTRCI = central circle; DCR = dark corner room; D= duration (s); D/F = duration per visit (s); F = frequency; L = latency (s); OCC = occurrence; PC = photocell; SAP = stretched attend posture; TOTACT = total activity, i.e. the sum of all frequencies; TOTARENA = total arena; TOTCORR = total corridor, i.e. the sum of all corridors. ^+^ p < 0.05 compared to age 4 weeks, ° p < 0.05 compared to AFR same age,* p < 0.05 compared to MS15W same age (repeated measures ANOVA between AFR, MS15W and MS360W followed by Fisher’s LSD test or χ*^2^*-test for differences in occurrence); ^&^ p < 0.05 compared to MS15W same age, ^§^ p < 0.05 compared to MS360W same age, ^@^ p < 0.05 compared to MS15E same age (repeated measures ANOVA on MS group (MS15 or MS360) and intake group (E or W) followed by Fisher’s LSD test).
