# Supplementary material for: Effects of Rearing Conditions on Behaviour and Endogenous Opioids in Rats with Alcohol Access during Adolescence
Source: PLoS One. 2013 Oct 2;8(10):e76591. doi: 10.1371/journal.pone.0076591 (PMC3788749; doi:10.1371/journal.pone.0076591)
Supplement: Table S2 — Mean ir beta-endorphin (BEND) levels (fmol/mg tissue) ± SEM in the dissected brain areas in the different groups of rats. (DOCX) [file pone.0076591.s003.docx]

Table S2. Mean ir beta-endorphin (BEND) levels (fmol/mg tissue) ± SEM in the dissected brain areas in the different groups of rats.

|  | **MS15W** | **MS360W** | **MS15E** | **MS360E** |
| --- | --- | --- | --- | --- |
| **AL** | 8163 ± 727 | 8979 ± 693 | 9209 ± 957 | 8308 ± 480 |
| **NIL** | 20824 ± 4734 | 9024 ± 1817 | 18780 ± 2640 | 13293 ± 1767 |
| **Pit** | 12018 ± 1058 | 9726 ± 610° | 11889 ± 985 | 10230 ± 512° |
| **HT** | 42.5 ± 6.0 | 37.4 ± 2.7 | 34.3 ± 2.8 | 32.6 ± 1.9 |
| **Nac** | 11.3 ± 4.4 | 13.3 ± 7.0 | 7.92 ± 2.3 | 9.69 ± 3.0 |
| **Amy** | 5.67 ± 2.0 | 4.44 ± 0.5 | 3.81 ± 0.4 | 3.86 ± 0.3 |
| **PAG** | 16.6 ± 1.9 | 11.4 ± 1.2°* | 13.6 ± 1.0 | 10.7 ± 0.8° |

MS15 = maternal separation 15 min, MS360 = maternal separation 360 min, E = ethanol, W = water, AL = anterior lobe of the pituitary, NIL = neurointermediate lobe of the pituitary, Pit = pituitary, HT = hypothalamus, Nac = nucleus accumbens, Amy = amygdala, PAG = periaqueductal gray area. ° *p* < 0.05 for all MS15 compared to all MS360 independent of intake, * *p* < 0.05 compared to MS15W (two-way factorial ANOVA followed by Fisher’s LSD test).
