# Supplementary material for: Effects of Rearing Conditions on Behaviour and Endogenous Opioids in Rats with Alcohol Access during Adolescence
Source: PLoS One. 2013 Oct 2;8(10):e76591. doi: 10.1371/journal.pone.0076591 (PMC3788749; doi:10.1371/journal.pone.0076591)
Supplement: Table S3 — Mean ir dynorphin B (DYNB) levels (fmol/mg tissue) ± SEM in the dissected brain areas in the different groups of rats. (DOCX) [file pone.0076591.s004.docx]

Table S3. Mean ir dynorphin B (DYNB) levels (fmol/mg tissue) ± SEM in the dissected brain areas in the different groups of rats.

|  | **MS15W** | **MS360W** | **MS15E** | **MS360E** |
| --- | --- | --- | --- | --- |
| **AL** | 36.0 ± 5.7 | 31.3 ± 3.7 | 34.9 ± 4.5 | 25.3 ± 2.5 |
| **NIL** | 572 ± 66 | 442 ± 64 | 521 ± 40 | 455 ± 31 |
| **HT** | 41.7 ± 3.5 | 36.6 ± 3.5 | 40.0 ± 2.7 | 39.9 ± 2.4 |
| **FCx** | 3.15 ± 0.3 | 3.66 ± 0.2 | 3.39 ± 0.2 | 3.43 ± 0.3 |
| **MPFCx** | 2.65 ± 0.3 | 2.58 ± 0.3 | 2.64 ± 0.2 | 2.73 ± 0.3 |
| **NAc** | 23.0 ± 3.3 | 28.1 ± 4.0 | 24.8 ± 3.3 | 23.7 ± 2.8 |
| **Str** | 10.8 ± 0.8 | 11.5 ± 0.6 | 11.1 ± 0.5 | 11.4 ± 0.7 |
| **HC** | 10.7 ± 1.0 | 11.1 ± 0.9 | 11.8 ± 0.6^#^ | 12.7 ± 0.7^#^ |
| **Amy** | 8.12 ± 0.9 | 9.10 ± 0.8 | 8.37 ± 0.5 | 7.79 ± 0.4 |
| **SN** | 48.6 ± 5.3 | 49.8 ± 5.9 | 56.7 ± 5.3 | 56.4 ± 6.1 |
| **VTA** | 6.81 ± 0.7 | 8.62 ± 2.5 | 5.95 ± 0.8 | 8.19 ± 1.4 |
| **PAG** | 6.82 ± 0.8 | 5.98 ± 0.7 | 5.57 ± 0.5 | 5.32 ± 0.5 |

MS15 = maternal separation 15 min, MS360 = maternal separation 360 min, E = ethanol, W = water, AL = anterior lobe of the pituitary, NIL = neurointermediate lobe of the pituitary, HT = hypothalamus, FCx = frontal cortex, MPFCx = medial prefrontal cortex, Nac = nucleus accumbens, Str = dorsal striatum, HC = hippocampus, Amy = amygdala, SN = substantia nigra, VTA = ventral tegmental area, PAG = periaqueductal gray area. ^#^ *p* < 0.05 for all ethanol-drinking MS rats compared to all water-drinking MS rats (two-way factorial ANOVA followed by Fisher’s LSD test).
