# Supplementary material for: Effects of Rearing Conditions on Behaviour and Endogenous Opioids in Rats with Alcohol Access during Adolescence
Source: PLoS One. 2013 Oct 2;8(10):e76591. doi: 10.1371/journal.pone.0076591 (PMC3788749; doi:10.1371/journal.pone.0076591)
Supplement: Table S4 — Mean ir Met-enkephalin-Arg6Phe7 (MEAP) levels (fmol/mg tissue) ± SEM in the dissected brain areas in the different groups of rats. (DOCX) [file pone.0076591.s005.docx]

Table S4. Mean ir Met-enkephalin-Arg^6^Phe^7^ (MEAP) levels (fmol/mg tissue) ± SEM in the dissected brain areas in the different groups of rats.

|  | **MS15W** | **MS360W** | **MS15E** | **MS360E** |
| --- | --- | --- | --- | --- |
| **AL** | 1.62 ± 0.6 | 1.66 ± 0.8 | 0.98 ± 0.1 | 0.74 ± 0.1 |
| **NIL** | 1.48 ± 0.2 | 2.11 ± 0.4 | 1.72 ± 0.3 | 1.88 ± 0.2 |
| **HT** | 73.4 ± 7.9 | 65.8 ± 6.9 | 67.2 ± 7.3 | 70.0 ± 6.5 |
| **FCx** | 4.18 ± 0.4 | 5.24 ± 0.4 | 4.20 ± 0.3 | 4.69 ± 0.6 |
| **MPFCx** | 5.79 ± 1.2 | 5.05 ± 0.6 | 5.10 ± 0.5 | 5.08 ± 0.6 |
| **Nac** | 68.3 ± 7.6 | 64.1 ± 6.2 | 66.7 ± 5.0 | 70.5 ± 4.9 |
| **Str** | 52.8 ± 5.4 | 53.6 ± 7.8 | 50.4 ± 5.2 | 52.7 ± 5.7 |
| **HC** | 5.81 ± 0.8 | 5.69 ± 0.5 | 5.85 ± 0.4 | 5.92 ± 0.3 |
| **Amy** | 24.5 ± 3.4 | 29.7 ± 3.3 | 34.9 ± 3.4^#^* | 33.0 ± 2.0^#^ |
| **SN** | 6.55 ± 1.1 | 6.52 ± 0.7 | 7.03 ± 0.8 | 7.33 ± 0.8 |
| **VTA** | 15.4 ± 2.0 | 13.7 ± 2.5 | 16.3 ± 1.9 | 14.3 ± 1.3 |
| **PAG** | 23.4 ± 4.7 | 21.3 ± 2.9 | 28.8 ± 3.0 | 24.1 ± 2.9 |

MS15 = maternal separation 15 min, MS360 = maternal separation 360 min, E = ethanol, W = water, AL = anterior lobe of the pituitary, NIL = neurointermediate lobe of the pituitary, HT = hypothalamus, FCx = frontal cortex, MPFCx = medial prefrontal cortex, Nac = nucleus accumbens, Str = dorsal striatum, HC = hippocampus, Amy = amygdala, SN = substantia nigra, VTA = ventral tegmental area, PAG = periaqueductal gray area. ^#^ *p* < 0.05 for all ethanol-drinking MS rats compared to all water-drinking MS rats, * *p* < 0.05 compared to MS15W (two-way factorial ANOVA followed by Fisher’s LSD test).
